# Substantially Improving CO_2_ Permeability and CO_2_/CH_4_ Selectivity of Matrimid Using Functionalized-Ti_3_C_2_T_*x*_

**DOI:** 10.1021/acsami.4c17315

**Published:** 2025-01-02

**Authors:** Mohammad Mozafari, Saeed Khoshhal Salestan, Ahmad Arabi Shamsabadi, Kritika Jha, Manushree Tanwar, Hyehyun Kim, Zahra Fakhraai, Masoud Soroush

**Affiliations:** † Department of Chemical and Biological Engineering, Drexel University, Philadelphia, Pennsylvania 19104, United States; ‡ Department of Chemical Engineering, Babol Noshirvani University of Technology, Babol 47148-71167, Iran; § Department of Mechanical Engineering, Donadeo Innovation Center for Engineering, Advanced Water Research Lab (AWRL), University of Alberta, Edmonton, Alberta 10-367 T6G 1H9, Canada; ∥ Department of Chemistry, University of Pennsylvania, Philadelphia, Pennsylvania 19104, United States; ⊥ Department of Materials Science and Engineering, Drexel University, Philadelphia, Pennsylvania 19104, United States

**Keywords:** mixed-matrix membrane, Ti_3_C_2_T_*x*_ MXene, surface functionalization, matrimid, gas separation

## Abstract

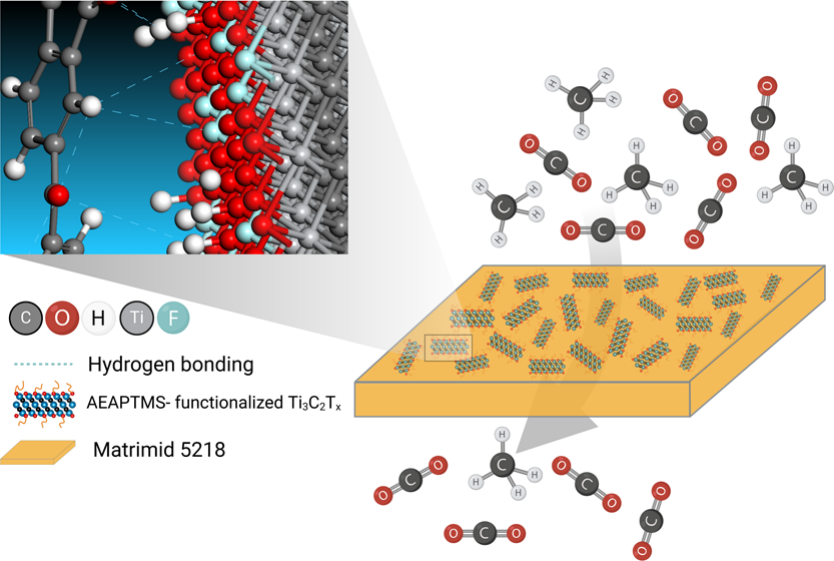

Mixed-matrix membranes (MMMs) with favorable interfacial interactions between dispersed and continuous phases offer a promising approach to overcome the traditional trade-off between permeability and selectivity in membrane-based gas separation. In this study, we developed free-standing MMMs by embedding pristine and surface-modified Ti_3_C_2_T_*x*_ MXenes into Matrimid 5218 polymer for efficient CO_2_/CH_4_ separation. Two-dimensional Ti_3_C_2_T_*x*_ with adjustable surface terminations provided control over these critical interfacial interactions. Characterization (Raman spectroscopy, XPS, DSC, FTIR) indicated the formation of hydrogen bonds between the termination groups on Ti_3_C_2_T_*x*_ and the carbonyl groups of Matrimid, promoting enhanced compatibility and dispersion of MXenes within the polymer matrix. The resulting MMMs with 5 wt % Ti_3_C_2_T_*x*_ showed a 67% increase in CO_2_ permeability and an 84% enhancement in CO_2_/CH_4_ selectivity compared to pristine Matrimid membranes. Surface modification of Ti_3_C_2_T_*x*_ using [3-(2-aminoethylamino)propyl]trimethoxysilane (AEAPTMS) further enhanced compatibility, leading to MMMs with 140% higher CO_2_ permeability and 130% greater CO_2_/CH_4_ selectivity. Molecular simulations suggested that AEAPTMS functionalization improved interfacial interactions with Matrimid chains, increasing the affinity of MXenes toward CO_2_ molecules. Additionally, the elongation of gas pathways, polymer chain disruption, and the presence of interlayer nanogalleries contributed positively to the enhanced separation performance. This work provides insights into tailoring nanomaterial–polymer interfaces to address the challenges of gas separation, paving the way for environmentally friendly CO_2_ separation technologies.

## 1 Introduction

Membranes are widely used in various industries for applications such as helium recovery from natural gas, hydrogen recovery, oxygen and nitrogen separation from air. Polymeric membranes are the commercially standard in these processes due to their excellent processability, low cost, simplicity, and high energy efficiency.^1,2^ However, despite their extensive use, polymeric membranes face challenges such as permeability-selectivity trade-off, swelling, physical aging, and plasticization, which hinder their separation performance and limit their industrial applicability.^3−5^ On the other hand, while inorganic membranes have surpassed the 2008 Robeson upper bound in separation performance, their application in commercial settings is hampered by concerns in scaling up for large modules and higher cost.^6^ To combine the advantages of both inorganic and polymeric materials, mixed-matrix membranes (MMMs) have been developed as the next generation of membranes.^7^ However, high particle loadings in MMMs can lead to agglomeration and the formation of nonselective interfacial microvoids, significantly reducing separation performance.^8^ To prepare efficient MMMs, several key parameters must be considered: (i) ensuring chemical compatibility at the interface between the dispersed (filler) and continuous (polymer) phases, (ii) achieving a homogeneous dispersion of fillers within polymer matrices, avoiding agglomeration, and (iii) maintaining well dispersity of filler dispersion in solvents used for making casting solutions.^9^ To address these issues, two-dimensional (2D) nanosheets have presented great potential for incorporation into polymer matrices due to their high hydrophilicity, flexibility, dispersibility, rich chemistry, and excellent structural properties.^10,11^ Embedding 2D nanosheets into polymer matrices not only provides nanochannels for enhanced gas molecule transport but also reduces polymer chain mobility.^12^

Transition metal carbides, nitrides, and carbonitrides, collectively known as MXenes, represent a relatively young family of 2D nanomaterials, typically formulated as M_*n*+1_X_*n*_T_*x*_ (*n* = 1–4). MXenes are primarily synthesized through the selective etching of A-group layers, predominantly from elements in IIIA or IVA, derived from precursor materials with the general formula M_*n*+1_AX_*n*_. Here, M represents an early transition metal such as Ti, V, Mo, Ta, Zr, etc., X denotes carbon and/or nitrogen, and T_*x*_ indicates surface termination groups, including −O, −OH, −F, and −Cl that are formed during the etching and delamination processes.^13−15^ The −OH terminations on MXene surfaces enable further surface functionalization using different agents,^16,17^ which can enhance their interfacial interactions with polymer chains. MXenes have promising characteristics, such as strong interactions with various solvents and tunable surface terminations due to their rich surface chemistry, polarity, and hydrophilicity,^18^ making them great candidates for fabricating MMMs. For instance, Wang et al. successfully integrated MXene nanosheets into a PIM-1 polymer matrix using a facile solution casting method to enhance CO_2_/N_2_ separation in MMMs.^19^ The polar functionalities of MXene improved CO_2_ uptake, while the interlayer spacing (∼0.35 nm) modulated the diffusion channels for selective gas separation. The resulting membranes exhibited a synergistic effect of solution-diffusion and molecular sieving mechanisms, achieving a CO_2_/N_2_ selectivity of 32.7 and a CO_2_ permeability of 12,475.3 Barrer, surpassing the 2019 Robeson upper bound. These results highlight the potential of MXene-based MMMs for industrial CO_2_ capture applications.

Previous studies have shown that incorporating MXenes into rubbery polymers significantly improves gas separation performance compared to pure membranes, thanks to the excellent compatibility between MXenes and this kind of polymers.^20,21^ However, to the best of our knowledge, the impact of interfacial interactions between MXenes and glassy polymers on the performance of their corresponding MMMs for gas separation applications remains not well understood. In this study, we investigate how enhanced compatibility between Ti_3_C_2_T_*x*_ MXene and Matrimid 5218, a glassy polyimide rich in functional groups, influences the CO_2_ transport properties of their corresponding MMMs. Matrimid 5218 (referred to as Matrimid in this study) was selected for its high gas selectivity, excellent thermal and mechanical properties, and versatile chemistry. Moreover, MXene-based dispersions have demonstrated long-term dispersity in polar organic solvents such as *N*-methyl-2-pyrrolidone (NMP) and *N*,*N*-dimethylformamide (DMF), which are commonly used to prepare polyimide casting solutions liked those used in membrane fabrication studies, due to their high polarity, dielectric constants, and solvent–surface interactions, as well as surface tension compatibility.^18^ It is hypothesized that embedding Ti_3_C_2_T_*x*_ nanosheets into Matrimid 5218 leads to the creation of CO_2_-selective nanochannels. To enhance the interfacial interactions between the MXene and Matrimid matrix, the Ti_3_C_2_T_*x*_ nanosheets were functionalized by [3-(2-aminoethylamino) propyl] trimethoxysilane (AEAPTMS) using a procedure from our previous study^16^ before incorporating them into Matrimid. This modification is also expected to prevent the formation of nonselective microvoids at the Matrimid/MXene interface.^22^ The presence of AEAPTMS is anticipated to mitigate the rigidification and pore blockage of polymer chains, thereby enabling optimal spacing between the polymer chains and Ti_3_C_2_T_*x*_ nanosheets. To validate these hypotheses, we employed a combination of molecular simulations, various characterization techniques, and gas permeation experiments.

## 2 Experimental and Computational Methods

### 2.1 Materials

Hydrochloric acid (HCl, ACS reagent, 37%), titanium carbide (TiC, 99.5%), titanium powder (Ti, 325 mesh 99.5%), and aluminum powder (Al, 325 mesh 99.5%) were obtained from Thermo Scientific. Lithium fluoride (LiF, >98%) and DMF(ACS grade, >99.9%) were supplied by BeanTown Chemical. AEAPTMS was purchased from Alfa Aesar. Glacial acetic acid (100%) was provided by BDH Co. CO_2_ and CH_4_ gases, both with >99.99% purity, were sourced from Airgas. Ethanol (>99.5%, ACS grade) was supplied by Millipore Sigma. Matrimid 5218 was purchased from PolyK Technologies. Celgard film (0.064 μm pore size, 3501 coated PP) was obtained from Celgard LLC Co. Deionized (DI) water (Millipore water, 18.2 MΩ) was used in all experiments. All chemicals were used as received.

### 2.2 Synthesis of Ti_3_AlC_2_ MAX

To synthesize the Ti_3_AlC_2_ MAX phase, TiC, Ti, and Al powders were combined in a 2:1:1 molar ratio and jar-milled in Nalgene high-density polyethylene bottles with yttria-zirconia balls at a 2:1 ball-to-powder mass ratio for 18 h at 64 rpm. The resulting mixture was then transferred into an alumina crucible and covered with graphite foil. The crucible was placed in a Carbolite Gero furnace (model 1700 °C) for reactive sintering. The temperature was ramped up to 1400 °C at a rate of 3.5 °C/min under an argon atmosphere (99.999% purity) and held for 4 h. Afterward, the furnace was cooled to room temperature at a rate of 10 °C/min. The sintered block was subsequently milled into a fine powder (<71 μm) using a TiN-coated milling bit.

### 2.3 Synthesis of Ti_3_C_2_T_*x*_ MXene

**Figure 1:**
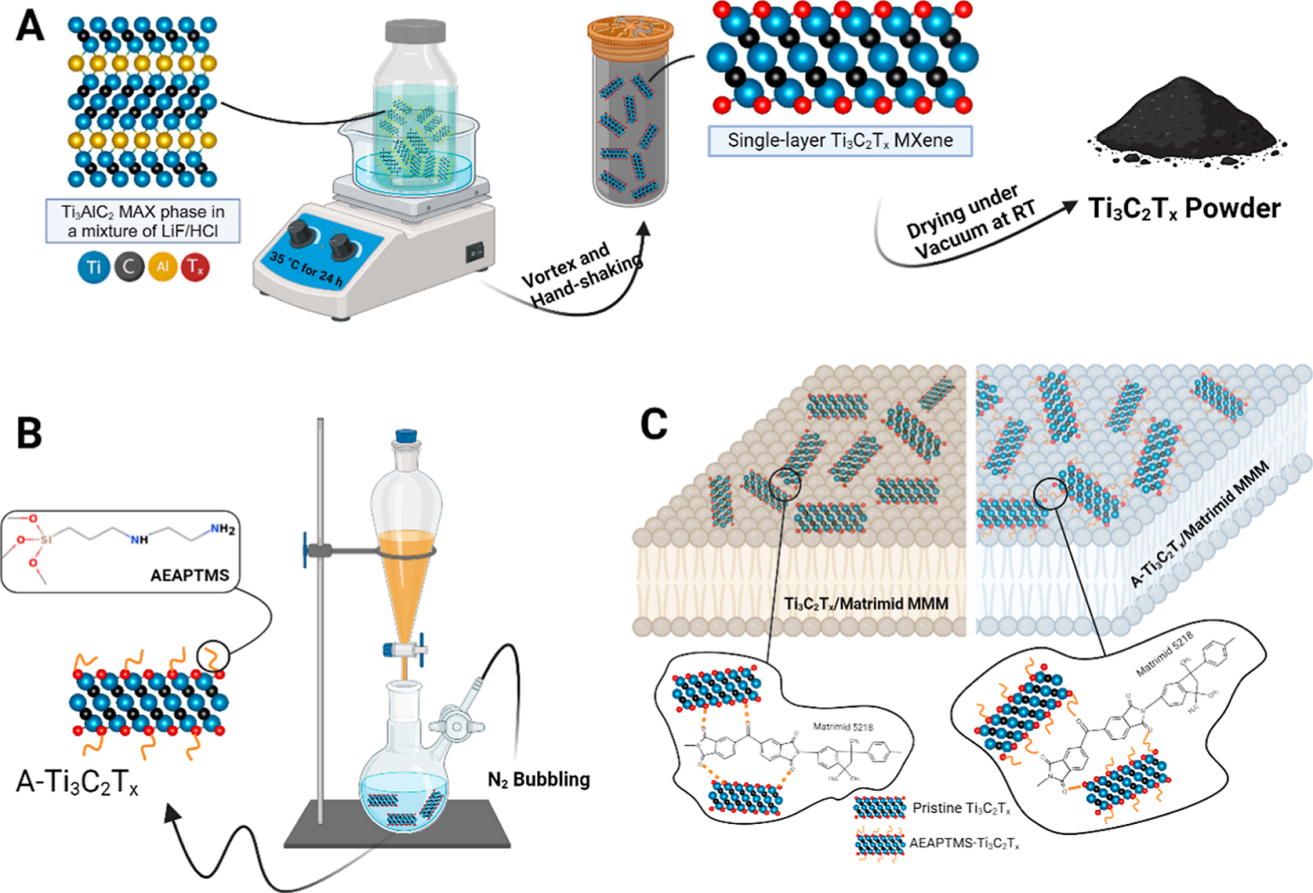
Schematic illustration of (A) selective etching of the Al layer using MILD route to synthesize Ti_3_C_2_T_*x*_ powder (B) functionalization of Ti_3_C_2_T_*x*_ MXene surface with AEAPTMS, and (C) proposed interfacial interactions between pristine Ti_3_C_2_T_*x*_ and Matrimid chains in Ti_3_C_2_T_*x*_/Matrimid MMMs, as well as the interactions between amine groups of the functionalized Ti_3_C_2_T_*x*_ and Matrimid chains in the A-Ti_3_C_2_T_*x*_/Matrimid MMM.

Ti_3_C_2_T_*x*_ MXene was synthesized from its precursor Ti_3_AlC_2_ MAX phase (Figure 1A) using the minimally intensive layer delamination (MILD) method.^23^ The etchant solution was prepared by mixing 1.6 g of LiF with 20 mL of 9 M HCl, followed by continuous stirring at 200 rpm for 5 min. Next, Ti_3_AlC_2_ MAX powder was slowly added to the etchant solution at a rate of 1 g/min. Once all MAX powder had been added, the temperature was set to 35 °C, and the stirring speed was increased to 400 rpm, allowing the reaction to proceed for 24 h. Once the reaction was complete, the mixture was transferred to a centrifuge tube and repeatedly washed with DI water at 3234 RCF for 5 min until the pH reached approximately 6. To exfoliate the MXene clay into single-to-few layer MXene, the resulting solution was vortexed for 30 min and then centrifuged at 2380 RCF for 30 min. The resulting multilayer Ti_3_C_2_T_*x*_ MXene colloidal solution was filtered using a Celgard film and dried under vacuum at room temperature to obtain Ti_3_C_2_T_*x*_ MXene powder.

### 2.4 Surface Functionalization of Ti_3_C_2_T_*x*_ MXene

A water/ethanol mixture (10/90 wt %) was employed to facilitate the surface functionalization of Ti_3_C_2_T_*x*_ MXene, providing the necessary water for the hydrolysis of AEAPTMS (Figure 1B). The reaction was conducted under continuous stirring (600 rpm) and nitrogen bubbling at room temperature for 8 h. Initially, a portion of required ethanol was added to the aqueous colloid of Ti_3_C_2_T_*x*_, followed by acetic acid to adjust the pH to 3.5. AEAPTMS, in a mass ratio of 2:1 to Ti_3_C_2_T_*x*_ MXene, was then dissolved in the remaining ethanol and gradually added dropwise to the reaction medium over the first 2 h. The resulting suspension was repeatedly washed with ethanol at 3500 rpm for 5 min to remove any unreacted substances. The final surface-modified Ti_3_C_2_T_*x*_, denoted as A-Ti_3_C_2_T_*x*_, was vacuum filtered onto a Celgard film and dried in a vacuum oven at 60 °C for 24 h to obtain free-standing films.

### 2.5 Preparation of Pristine Matrimid and Mixed-Matrix Membranes

Pristine Matrimid membranes and MMMs containing varying concentrations of Ti_3_C_2_T_*x*_ MXene were prepared following a previously established protocol.^24^ A 10 wt % solution of pristine Matrimid was prepared by gradually dissolving 0.8 g of Matrimid in 7.2 g of DMF in three steps. To fabricate the MMMs, specific amounts of pristine Ti_3_C_2_T_*x*_ (0.2, 0.5, 0.8, and 1 wt %) and A-Ti_3_C_2_T_*x*_ (0.5, 0.8, and 1 wt %) were dispersed in DMF. Following filtration, dried Ti_3_C_2_T_*x*_ and A-Ti_3_C_2_T_*x*_ samples were obtained in the form of films. Before redispersion in DMF, these films were carefully broken into smaller pieces. The resulting mixtures were bath sonicated for 3 h to ensure uniform dispersion and reduce the size of nanosheets. To ensure uniform exposure to ultrasound power, the vials were rotated periodically during the sonication process. Subsequently, Matrimid was then added to the suspension in the same stepwise manner. All polymeric dope solutions were continuously stirred for 24 h. After degassing, the dope solutions were cast into Petri dishes and placed in a vacuum oven at 70 °C for 24 h to remove the solvent. The compositions of each polymeric dope solution comprising Matrimid and MXenes are listed in Table S1. Pristine Matrimid membranes and Ti_3_C_2_T_*x*_-Matrimid MMMs were labeled as PM and MM-α, respectively, where α represents the weight percentage of pristine Ti_3_C_2_T_*x*_ (α = 2, 5, 8, 10) within the polymeric phase of each membrane. For example, MMMs containing 5, 8, and 10 wt % of A-Ti_3_C_2_T_*x*_ is labeled as A-MM-5, A-MM-8, and A-MM-10, respectively. Figure 1C shows a schematic illustration of the proposed interfacial interactions between pristine Ti_3_C_2_T_*x*_ and Matrimid chains in Ti_3_C_2_T_*x*_/Matrimid MMMs, as well as the interactions between amine groups of the functionalized Ti_3_C_2_T_*x*_ and Matrimid chains in the A-Ti_3_C_2_T_*x*_/Matrimid MMM.

### 2.6 Characterizations

Ti_3_C_2_T_*x*_ MXene, modified MXene, and fabricated membranes were characterized using Fourier transform infrared spectroscopy (FTIR) in attenuated total reflectance (ATR) mode, performed on a Thermo Fisher Nicolet iS50 FTIR Spectrometer (USA) within the frequency range of 400–4000 cm^–1^. Thermogravimetric analysis (TGA) was conducted on a TA Instruments Q50, heating the samples from 25 to 600 °C at a rate of 10 °C/min under Ar flow (60 mL/min). Differential scanning calorimetry (DSC) analysis was conducted using a TA Instruments Q20 calorimeter, scanning from room temperature to 500 °C at a rate of 10 °C/min under an Ar atmosphere. Dynamic light scattering (DLS) was performed using a NanoBrook ZetaPALS instrument (Brookhaven Instrument, USA). Raman spectroscopy was utilized to validate the presence of Ti_3_C_2_T_*x*_ MXene within the polymeric phase. These experiments were performed on a JASCO NRS-5500 micro-Raman spectrometer using a 100× objective lens and laser wavelength of 785 nm at 1.3 mW laser power and grating size of 300 grooves per mm. Each Raman spectrum was taken as the average of two accumulations of 120 s each. The morphology of Ti_3_C_2_T_*x*_ MXene and the prepared membranes was analyzed by scanning electron microscopy (SEM) using an Apreo 2S Low Vac system (ThermoFisher). Additionally, energy dispersive X-ray spectroscopy (EDS) with ChemiSEM Technology was employed for elemental imaging. X-ray photoelectron spectroscopy (XPS) was utilized to characterize the chemical composition of A-Ti_3_C_2_T_*x*_ MXene and the prepared membranes, performed with a PHI VersaProbe 5000 Physical Electronics XPS system (USA) equipped with a 200 μm and 50 W monochromatic Al Kα (1486.6 eV) X-ray source. Mechanical properties of the membranes were analyzed using dynamic mechanical analysis (DMA) at room temperature on an RSA-G2 instrument. X-ray diffraction (XRD) was conducted on both pure and functionalized Ti_3_C_2_T_*x*_ MXene using a Rigaku SmartLab system with a Cu Kα anode, operating at 40 mA and 40 kV within the 2θ range of 3–70°. Atomic force microscopy (AFM) imaging was carried out using a Bruker Icon (Bruker, Santa Barbara, CA) instrument with a tapping frequency of 300 kHz.

### 2.7 Molecular Simulations

Favorable interactions between the filler and polymer matrix enable interfacial integrity to avoid nonselective microvoid formation. To assess the interaction potential between Ti_3_C_2_T_*x*_ MXenes and Matrimid and investigate the effect of functional groups on these interactions, molecular simulations were performed using Materials Studio software and the universal force field (UFF) widely used for MXene structures.^23^ To consider intermolecular forces during the simulation, “Charge Qeq” was used to set the electrostatic charge of atoms, and Ewald and atom-based summation methods were utilized for electrostatic (with an accuracy of 10^–5^ kcal/mol) and van der Waals interaction (cutoff distance is 18 Å), respectively.

To prepare the structures, the crystal structure of Ti_3_C_2_T_2_ (T = O, F, and OH) monosheets was first rebuilt based on the information provided in previous work by Hu et al.^25^ They implemented density functional theory (DFT) calculation using the cambridge sequential total energy package (CASTEP)^26,27^ to obtain the optimized unit cell for different MXene samples. Their simulations were concluded with crystal structures, all of which possessed the crystal space group of *P*3̅*m*1 (no. 164), indicating hexagonal (γ = 120°) unit cells. The detailed summary of the unit cells they achieved, including the positions of central and surface Ti atoms (Ti1 and Ti2, respectively) are reported in Table S2 of Supporting Information.

Next, a supercell was generated by expanding the unit cell in the *x* and *y* directions to obtain MXene monosheets. Then, the nanosheets were used to make vacuum slabs, as depicted in Figure S1 of Supporting Information. The length of vacuum space was adjusted to 60 Å to eliminate the effect of boundary conditions in the *z* direction. Vacuum slab with Ti_3_C_2_(OH)_2_ was chosen to be functionalized with the aminosilane agent (AEAPTMS). The structure of functionalized MXene was then optimized through the “geometry optimization” module in the software.^23^ After that, the atoms on the MXene surface, which is to undergo an annealing process, were targeted as adsorption regions to find the lowest energy configuration of the Matrimid’s repeating unit nearby. The concept of simulated annealing comprises a heating process to a high temperature followed by a very slowly cooling down process in a controlled manner to reach a global minimum energy level while every single step is approximately in a thermodynamic equilibrium.^28,29^ During the heating–cooling process the temperature never exceeded 10^5^ K, and the final temperature was also 10 K where the number of annealing cycles was set to 5 and 100,000 steps were taken for each cycle. The simulation approach applies a canonical Monte Carlo sampling of the configuration space of the adsorbate–adsorbent system during which the system is gradually cooled down. In each step, the current state is altered by a randomly selected neighboring state in which if the energy decreases it is an acceptable state and is defined as the starting point for the next step otherwise it undergoes a decision-making process whether the new state can be accepted. How accepting the state of higher energy is, completely depends on the energy difference between the states and the temperature of the system. It is noteworthy that the applied method allows the system to explore states that have higher energy, leading to escape from the local minimum and providing better search results.

### 2.8 Gas Permeation Experiments

A constant volume/variable pressure setup was utilized to measure the gas permeation properties of each membrane, as depicted in Figure S2. The system is equipped with a Millipore membrane holder (XX4502500, effective area of 2.2 cm^2^), a gas line, and a vacuum line to maintain the permeate side under vacuum (2 × 10^–3^ Torr). This configuration enables us to monitor pressure changes over time using a Pressure Logger (Vacuum Track-It, 760 to 0 Torr, Monarch Instrument). Gas permeability (*P*_*i*_) (1 Barrer = 1 × 10^–10^ cm^3^ (STP) cm cm^–2^ s^–1^ cmHg^–1^) is calculated using^20^
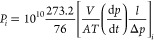
1where *A* is the effective membrane area (cm^2^), *T* is the temperature (K), Δ*p* is the pressure difference across the membrane (cmHg), *l* is the membrane thickness (cm), V is the volume of the downstream side (50 cm^3^), and  is the rate of change of permeate pressure (cmHg) when the pressure changes linearly with time (s). Additionally, the ideal gas selectivity (α_*i*/*j*_) is calculated using
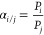
2where *P*_*i*_ and *P*_*j*_ are the permeabilities of the gas pair. The time lag method is used to determine the diffusivity coefficients (*D*_*i*_)
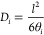
3where θ_*i*_ denotes the time lag (also known as delay time) in seconds obtained from a pressure-time curve. Gas transport through self-standing membranes is assumed to follow the solution-diffusion mechanism,^30^ where the permeability of each gas through the polymeric membrane is determined by multiplying the gas diffusivity coefficient (measured in 10^–8^ cm^2^·s^–1^) and solubility coefficient (measured in 10^–2^ cm^3^ (STP)·cm^–3^·cmHg^–1^). Therefore, the solubility coefficient (*S*_*i*_) can be determined as follows
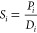
4

## 3 Results and Discussion

### 3.1 Characterization of Ti_3_C_2_T_*x*_ and Modified Ti_3_C_2_T_*x*_ MXenes

**Figure 2:**
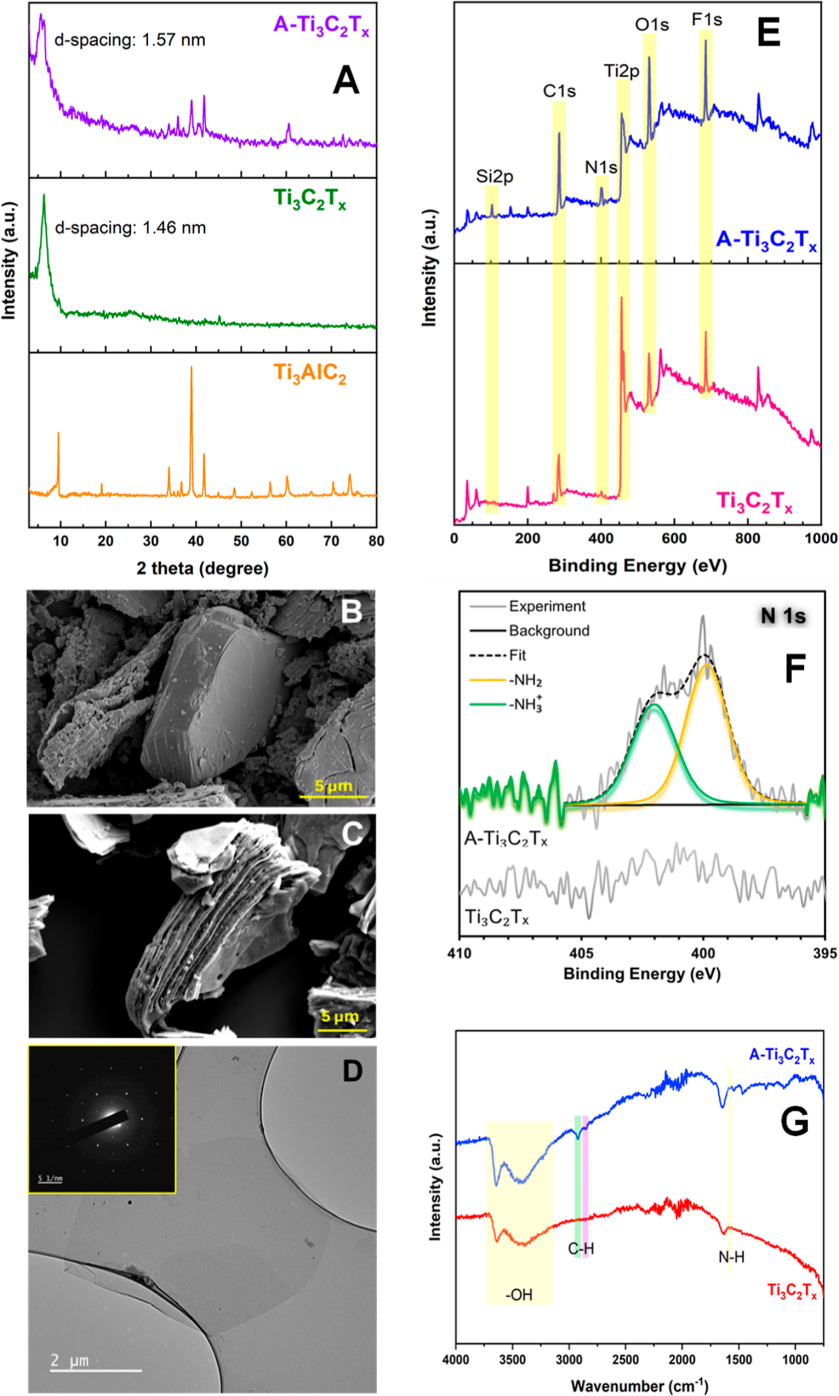
Characterization of Ti_3_AlC_2_ MAX phase, Ti_3_C_2_T_*x*_, and A-Ti_3_C_2_T_*x*_. (A) XRD patterns of Ti_3_AlC_2_, Ti_3_C_2_T_*x*_, and A-Ti_3_C_2_T_*x*_. SEM images of (B) Ti_3_AlC_2_ and (C) Ti_3_C_2_T_*x*_. (D) TEM image of Ti_3_C_2_T_*x*_ nanosheets (inset shows the selected-area electron diffraction (SAED) patterns at a high resolution). (E) XPS survey of A-Ti_3_C_2_T_*x*_ (top) and Ti_3_C_2_T_*x*_ (bottom). Yellow highlights indicate the peaks corresponding Ti 2p, C 1s, O 1s, Si 2p, and N 1s. (F) High-resolution N 1s XPS spectra along with the best fit (dashed line) for A-Ti_3_C_2_T_*x*._ (G) FTIR spectra of Ti_3_C_2_T_*x*_ and A-Ti_3_C_2_T_*x*_. Highlighted regions show the absorption bands corresponding to −OH, C–H, and N–H bonds.

Ti_3_C_2_T_*x*_ was synthesized using a MILD etching method using a mixture of LiF and HCl to produce MXene layers ranging from single to a few layers with minimal defects.^31^ More details can be found in the Experimental and Computational Methods section. The crystallinity of Ti_3_AlC_2_ MAX phase, pristine Ti_3_C_2_T_*x*_, and A-Ti_3_C_2_T_*x*_ MXenes was evaluated using XRD, as depicted in Figure 2A. The disappearance of the diffraction peak corresponding to the (104) planes of the Ti_3_AlC_2_ MAX phase located around 39° after chemical etching indicated the successful removal of Al layers.^32^ Additional peaks observed between 20–40° for Ti_3_C_2_T_*x*_ MXenes confirmed the proper periodicity of MXene layers. Moreover, the shift in the diffraction peak corresponding to the (002) planes of the Ti_3_AlC_2_ MAX phase to lower angles indicates the introduction of water molecules and surface terminations, which expand the d-spacing between adjacent MXene layers from 0.94 to 1.46 nm.^33^ Following surface functionalization with AEAPTMS, the d-spacing of Ti_3_C_2_T_*x*_ MXenes increased further, as evidenced by the appearance of a peak at 5.59°, corresponding to an expanded d-spacing of 1.57 nm. SEM images in Figure 2B,C depict both the Ti_3_AlC_2_ MAX phase and Ti_3_C_2_T_*x*_ MXenes, showing the pristine Ti_3_C_2_T_*x*_ MXenes with a characteristic accordion-like structure resulting from the etching and removal of Al layers from the parent Ti_3_AlC_2_ MAX phase. In this structure, single-layer nanosheets are interconnected by weak van der Waals forces.

The multilayer Ti_3_C_2_T_*x*_ MXenes were further delaminated into single layers or a few layers through sonication, as detailed in the Experimental and Computational Methods section and our previous study.^23^ EDS and element mappings of multilayer Ti_3_C_2_T_*x*_ presented in Figure S3, confirm the uniform distribution of Ti, C, O, F, and Cl elements and the successful removal of Al layers. The Ti_3_C_2_T_*x*_ nanosheets were characterized using AFM, as shown in Figure S4. The height profile (inset) indicates that the average thickness of the nanosheets is 12.3 nm. Based on the d-spacing of Ti_3_C_2_T_*x*_, determined from XRD to be approximately 1.43 nm (including interlayer spacing and one Ti_3_C_2_T_*x*_ single layer), each nanosheet is estimated to consist of 8–9 layers. The TEM image (Figure 2D) of pristine Ti_3_C_2_T_*x*_ reveals that the delaminated nanosheet, representing a single layer, is transparent. High-resolution selected-area electron diffraction (SAED) patterns confirm the high crystallinity and hexagonal structure of the single layer, with no obvious carbide amorphization or nanoscale defects. Additionally, the folded edges of the flakes indicate their flexible nature.

The Raman spectrum of pristine Ti_3_C_2_T_*x*_, collected from 100 to 800 cm^–1^ (Figure S5), verifies the successful synthesis of Ti_3_C_2_T_*x*_. The peaks at 594 cm^–1^ (ω_4_), 386 cm^–1^ (ω_5_), and 284 cm^–1^ (ω_5_) correspond to the *E*_g_ group vibrations, which include in-plane modes of surface terminations, Ti, and C atoms.^25,34^ The peaks at 717 cm^–1^ (ω_3_) and 198 cm^–1^ (ω_2_) represent *A*_1g_ symmetry out-of-plane vibration of C and Ti atoms, respectively.^35^

The chemical state of the elements in pristine Ti_3_C_2_T_*x*_ and A-Ti_3_C_2_T_*x*_ were analyzed using XPS. The XPS survey spectrum, shown in Figure 2E, demonstrates that the synthesized Ti_3_C_2_T_*x*_ is mainly composed of Ti, C, O, and F atoms. In contracts, the survey spectrum of A-Ti_3_C_2_T_*x*_ shows two additional peaks corresponding to Si and N, confirming the successful surface functionalization of Ti_3_C_2_T_*x*_ with AEAPTMS. The presence of Si and N is attributed to AEAPTMS molecules, either through physical adsorption or covalent bonding, as any unreacted silane coupling agents were removed by washing the samples three times with ethanol.^16^

Figure S6A–D presents high-resolution XPS spectra of Ti_3_C_2_T_*x*_ (see Supporting Information, Table S3). The Ti 2p spectrum of Ti_3_C_2_T_*x*_ (Figure S6A) was analyzed using the Fit-V protocol, identifying five components: C–Ti–(O,O,O), C–Ti–(O,O,F), C–Ti–(O,F,F), C–Ti–(F,F,F), and TiO_2–*x*_F_2*x*_, with binding energies at 454.9, 455.8, 456.8, 458.2, and 459.4 eV, respectively.^36^ Although mild oxidation of MXene (TiO_2–*x*_F_2*x*_) cannot be completely avoided during chemical etching, this oxidation would be evident as a prominent peak in oxidized MXene samples, which is not observed here. The C 1s spectrum exhibits (Figure S6B) four distinct components, including C–Ti (281.8 eV), C–C (284.7 eV), C–O (286.0 eV), and O–C=O (288.7 eV). The deconvolution of the O 1s spectrum in Figure S6C identifies components corresponding to C–Ti–O (529.6 eV), TiO_2–*x*_F_2*x*_ (530.6 eV), C–Ti–OH/C–Ti–OH,F (531.9 eV), and adsorbed water (533.0 eV). In the F 1s region (Figure S6D), two distinct components are observed: C–Ti–F (685.0 eV) and F contaminations (686.7 eV). The protonation state and presence of amine groups on the A-Ti_3_C_2_T_*x*_ surface were confirmed by analyzing the N 1s high-resolution spectrum (Figure 2F), which was fitted to two peaks corresponding to protonated and free amines, centered at binding energies of 402.0 and 399.8 eV, respectively.^37^ The FTIR spectroscopy of Ti_3_C_2_T_*x*_ and A-Ti_3_C_2_T_*x*_ (Figure 2G) indicates the emergence of two distinct peaks after surface functionalization with AEAPTMS, at 2854 and 2923 cm^–1^. These peaks correspond to the asymmetric and symmetric vibrations of C–H bonds in the alkyl chains of AEAPTMS, respectively.^38^ Additionally, the bending mode of the N–H bond of the primary amine appears as a peak at 1580 cm^–1^.^39^ The broad peak observed around 3500 cm^–1^ is attributed to adsorbed water molecules and surface −OH terminations.

In our previous work, it was demonstrated that the lateral size of Ti_3_C_2_T_*x*_ significantly affects the separation performance of the membranes.^20^ Consequently, a 3 h sonication process was conducted to obtain Ti_3_C_2_T_*x*_ sheets with smaller lateral sizes. Figure S7A,B show the particle size distributions of Ti_3_C_2_T_*x*_ before and after, respectively. Both samples exhibit two distinct distributions in the intensity of the size distribution, with the prominent peak representing the primary particle size distribution. The 3 h sonication reduced the average particle size from 1780 to 223 nm and resulted in a narrower, more uniform particle size distribution. Notably, the full width at half-maximum (fwhm) of Ti_3_C_2_T_*x*_ decreased from 308.46 to 23.21 nm after sonication. For more detailed information on the impact of sonication on the MXene structure please refer to Supporting Note S1 in the Supporting Information. The redispersion stability of Ti_3_C_2_T_*x*_ and A-Ti_3_C_2_T_*x*_ in DMF was qualitatively evaluated over time (as-prepared, 24, 48, and 72 h). As shown in Figure S8, both pristine Ti_3_C_2_T_*x*_ and A-Ti_3_C_2_T_*x*_ formed stable dispersions immediately after bath sonication. The dispersion of pristine Ti_3_C_2_T_*x*_ in DMF (column B) remained highly stable even after 72 h, similar to the MXene suspension in DI water (column A). In contrast, after 72 h, sedimentation and a decrease in concentration were observed for A-Ti_3_C_2_T_*x*_ in DMF (column C). This strong dispersion stability can be attributed to factors such as the polarity of DMF, its high surface tension, high boiling point, and high dielectric constant.^18^ Furthermore, surface functionalization with AEAPTMS introduces silane groups that react with hydroxyl groups on the MXene surface. This reaction along with the steric hindrance provided by the functionalization agent minimizes π–π stacking between adjacent A-Ti_3_C_2_T_*x*_ nanosheets and prevents aggregation.^40^ The strong dispersion stability and steric effects of functionalization mitigate stacking tendencies, as observed in the absence of agglomerates in A-MM-5 membrane.

### 3.2 Characterization of Ti_3_C_2_T_*x*_–based Mixed Matrix Membranes

**Figure 3:**
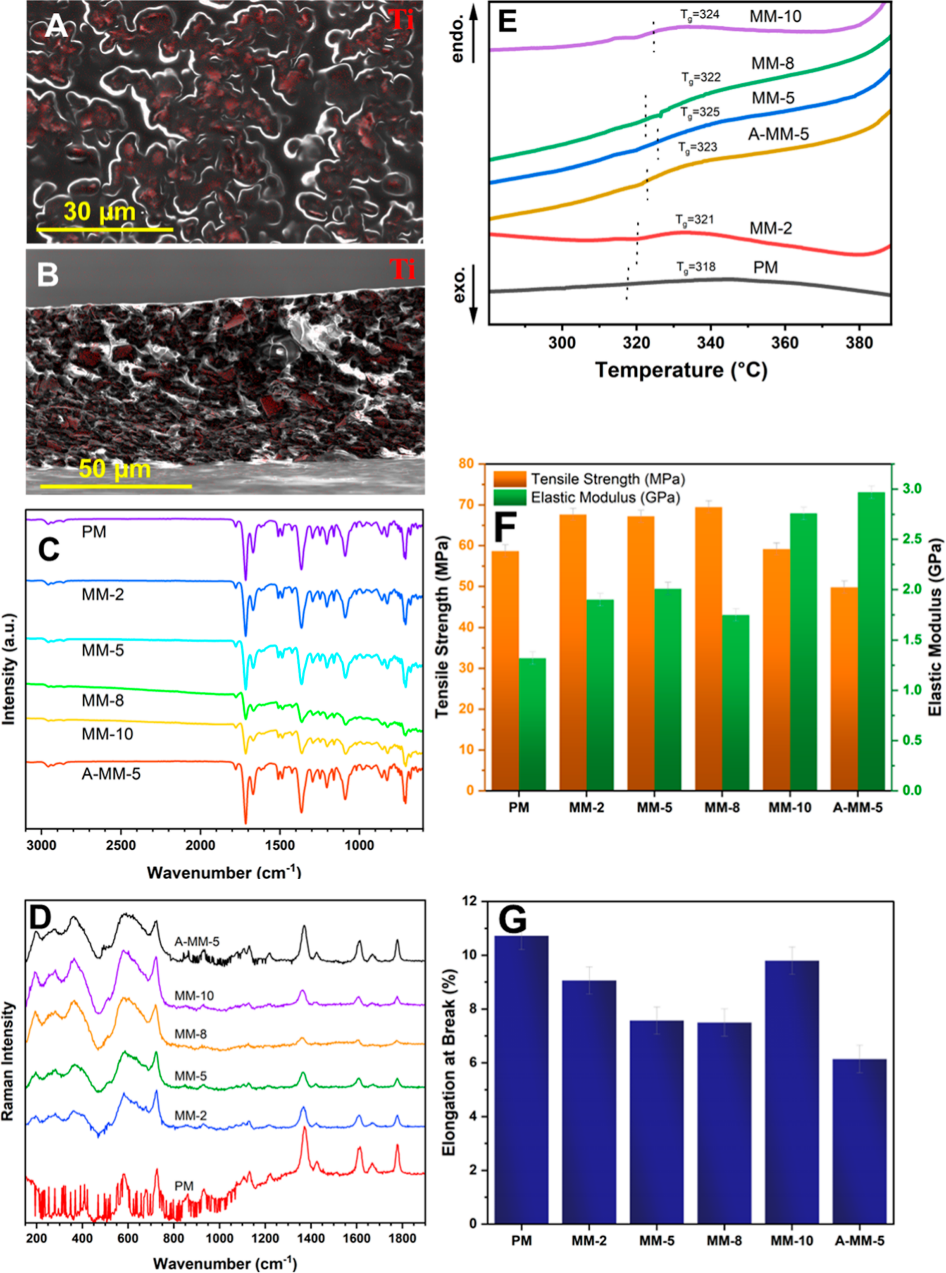
(A) Surface and (B) cross-sectional SEM images and corresponding EDS mapping of MM-5 membrane. Titanium (Ti) distribution is indicated by the red color. (C) FTIR spectra and (D) Raman spectra of the pristine Matrimid and Ti_3_C_2_T_*x*_-Matrimid membranes. (E) DSC curves of the pristine Matrimid and Ti_3_C_2_T_*x*_-Matrimid membranes at a heating rate of 10 °C/min. Mechanical properties: (F) tensile strength and elastic modulus and (G) elongation at break of the pristine Matrimid and Ti_3_C_2_T_*x*_-Matrimid membranes.

When designing MMMs, it is crucial to choose materials that not only stand out individually in CO_2_ separation but also have chemical affinities compatible with the inorganic inclusions. This compatibility ensures appropriate interphase adhesion, reducing the likelihood of nonselective microvoids formation, which can noticeably impair gas selectivity.^41^ In this context, MXene-based MMMs were prepared by embedding pristine Ti_3_C_2_T_*x*_ (2, 5, 8, and 10 wt %) and A-Ti_3_C_2_T_*x*_ (5, 8, 10 wt %) into Matrimid, as outlined in the Experimental and Computational Methods section. The effectiveness of interfacial interaction between MXenes and Matrimid chains can be physically confirmed using cross-sectional SEM images of the membranes. Figure S9A,B shows cross-sectional SEM images of PM (with a thickness of ∼86 μm) and MM-5 membranes, which has a thickness of ∼65 μm. As seen in Figure S9A, the pristine Matrimid membrane has a dense and uniform structure without defects. In contrast, the MM-5 membrane (Figures 3A,B and S9B) demonstrates a uniform dispersion of Ti_3_C_2_T_*x*_ within the polymer matrix, indicating favorable interactions between the polymer chains and MXene nanoflakes. EDS analysis from the surface and cross-sectional images of the MM-5 membrane further supports these findings (Figure 3A,B). The Ti element, a key component of MXene, is highlighted in red to indicate its presence throughout the membrane. The consistent distribution of Ti suggests a uniform dispersion of MXene within the polymer matrix. Additionally, the emergence of the network of reticular pattern morphology of the Matrimid (Figure S9B) further supports the indication of favorable interfacial interactions.^42,43^ However, as the Ti_3_C_2_T_*x*_ MXene loading increased to 8 and 10 wt %, the MM-8 and MM-10 membranes displayed nonselective galleries within the membranes (see Supporting Information, Figure S9C,D) due to agglomeration of the MXene nanoflakes. Therefore, membranes with 5 wt % loading of modified Ti_3_C_2_T_*x*_ were synthesized for further evaluation of MXene/Matrimid compatibility. Similarly, strong dispersion was observed in A-MM-5 containing A-Ti_3_C_2_T_*x*_ (Figure S9E). In contrast, MMMs with pristine Ti_3_C_2_T_*x*_ exhibited small agglomerations, which were absent in A-MM-5. The interfacial adhesion between MXene and Matrimid was also significantly enhanced in the case of A-Ti_3_C_2_T_*x*_ which contains −NH_2_ groups. This improved adhesion, caused by the presence of −NH_2_ groups, was also observed for PIM-1/UiO-66-NH_2_.^44^ As a result, SEM characterization showed the uniform dispersion of pristine Ti_3_C_2_T_*x*_ or A-Ti_3_C_2_T_*x*_ within the Matrimid matrix, with no observable microvoids or phase separation at lower loadings (e.g., 5 wt %). This suggests good interfacial adhesion between MXene and Matrimid, which is crucial for maintaining the structural integrity of the MMMs. While SEM provides indirect evidence of interfacial compatibility, complementary techniques such as FTIR, XPS, and Raman spectroscopy provide more direct confirmation of the interfacial interactions. A detailed discussion on the interfacial adhesion between A-Ti_3_C_2_T_*x*_ and Matrimid is provided later in this section.

To explore the specific interactions between the Matrimid matrix and pristine Ti_3_C_2_T_*x*_ or A-Ti_3_C_2_T_*x*_, FTIR, Raman, and XPS measurements were performed (Figures 3C,D and S10 of Supporting Information). As seen in Figure 3C, there is no significant difference between the FTIR spectra of pristine Matrimid and Ti_3_C_2_T_*x*_-Matrimid membranes (more details are provided in Supporting Note S2 in Supporting Information). The lack of notable changes in the absorption bands suggests substantial chemical interactions between the Matrimid chains and either pristine Ti_3_C_2_T_*x*_ or A-Ti_3_C_2_T_*x*_ are absent. It is also possible that even at 10% loading, the magnitude of such changes is negligible compared to other peaks. However, slight downshifts in the carbonyl group of the imide structure of Matrimid in Ti_3_C_2_T_*x*_-Matrimid and A-Ti_3_C_2_T_*x*_-Matrimid membranes may indicate the presence of physical interactions and hydrogen bonding between the incorporated MXenes and Matrimid chains, as previously reported.^45,46^ A similar behavior was observed in Ti_3_C_2_T_*x*_-Pebax membranes, attributed to the formation of hydrogen bonding between amide groups and Ti_3_C_2_T_*x*_ MXene.^20^ By comparing the Raman spectra of pristine Matrimid and Ti_3_C_2_T_*x*_-Matrimid membranes (Figure 3D), the presence of pristine Ti_3_C_2_T_*x*_ and A-Ti_3_C_2_T_*x*_ MXenes within the polymeric phase is corroborated, as intrinsic MXenes peaks are directly observed (Supporting Information, Figure S5) in the spectra of Ti_3_C_2_T_*x*_-Matrimid membranes. Raman spectroscopy also confirms the formation of physical interactions between Matrimid chains and Ti_3_C_2_T_*x*_ MXene, as the peaks related to Ti_3_C_2_T_*x*_ MXenes shift toward higher frequencies (Supporting Information, Table S4).^20^ The analysis of high-resolution XPS spectra of C and O atoms (see Figure S10 and Table S5 in Supporting Information) also verifies the formation of hydrogen bonds between Ti_3_C_2_T_*x*_ and modified Ti_3_C_2_T_*x*_, and Matrimid chains. The binding energies of C and O atoms slightly shift, indicating changes in their electron cloud densities. These changes can be attributed to the formation of hydrogen bonds between the surface terminations of pristine Ti_3_C_2_T_*x*_ (−OH) and modified Ti_3_C_2_T_*x*_ (−OH, and −NH_2_) and =O groups of Matrimid, as previously discussed.^20^

The glass transition temperature (*T*_g_), as measured by DSC, is the key indicator of interfacial interactions in MMMs. A shift in *T*_g_ toward higher temperatures indicates strong interactions between the polymer chains and fillers, resulting in increased rigidification and decreased polymer chain mobility.^47,48^ The DSC results, depicted in Figure 3E and listed in Table S6, illustrate a 3–6 °C change in *T*_g_ at different MXene loadings. The direction of change in *T*_g_ substantiates strong interactions, attributed to the formation of hydrogen bonds between Ti_3_C_2_T_*x*_ MXenes and Matrimid chains.^49,50^

The inclusion of MXenes in the Matrimid matrix can improve mechanical properties of the resulting MMMs. As shown in Figure 3F, even the embedding of 2 wt % of pristine Ti_3_C_2_T_*x*_ resulted in 45% improvement in the elastic modulus, indicating strong interactions between Matrimid chains and the MXene. Notably, the incorporation of 5 wt % of A-Ti_3_C_2_T_*x*_ led to a substantial improvement (∼125%) in elastic modulus, attributed to favorable interfacial interactions between the Matrimid chains and the −NH_2_ groups on A-Ti_3_C_2_T_*x*_ MXene. Fluctuations in tensile strength are also evident in Figure 3F (additional tensile curves can be found in Figure S11 in Supporting Information), with improvement observed for MM-2, MM-5, MM-8, and MM-10 membranes compared to the PM membrane. Generally, robust interfacial interactions between pristine Ti_3_C_2_T_*x*_ or A-Ti_3_C_2_T_*x*_ MXenes and the polymer can restrict polymer chain mobility, resulting in more rigid but more brittle MMMs. Furthermore, it is interesting to note that elongation at break decreases with increasing MXene loading (Figure 3G), indicating a more rigid structure due to the incorporation of pristine Ti_3_C_2_T_*x*_. Such phenomena are also observed in graphene oxide (GO)/Matrimid MMMs.^51^

The TGA results for pristine Matrimid and Ti_3_C_2_T_*x*_-Matrimid membranes are shown in Figure S12. In all cases, two distinct weight loss events are observed. The initial weight loss, occurring in the temperature range of 180–325 °C and accounting for approximately 9%, is attributed to the evaporation of residual solvents in the membranes. The subsequent significant weight loss, commencing at 450 °C, corresponds to the thermal degradation of the polymer matrix. Upon incorporation of Ti_3_C_2_T_*x*_ into the Matrimid matrix, a delay in the onset of thermal degradation is observed, beginning around 470 °C. This delay confirms the strong interfacial interactions between pristine Ti_3_C_2_T_*x*_ and Matrimid, resulting in enhanced thermal resistance of MMMs compared to the pristine Matrimid membrane.

To gain a deeper understanding of Matrimid-Ti_3_C_2_T_*x*_ interactions, molecular simulations were conducted to explore the formation of hydrogen bonds between Ti_3_C_2_T_*x*_ with different surface terminations and Matrimid’s repeating unit. Figure S13 illustrates the preferential configurations of the Matrimid’s repeating unit interacting with the MXene surface as determined by the simulation. The figure indicates that the lowest energy configuration can vary with changes in surface. As detailed in Table S7, further analysis revealed that the number of hydrogen bonds with a bond length of 3 Å or shorter increased when the MXene surface was functionalized by AEAPTMS. This indicates that the proportion of strong and moderate hydrogen bond interactions among the species increases with surface modification with this agent.^52^ Furthermore, the functionalization led to an increase in the number of hydrogen bonds ranging between 3 and 4 Å when Ti_3_C_2_(OH)_2_ was modified by the aminosilane agent, proving that the surface becomes more attractive for interaction with the polymer. This enhanced interaction keeps the polymer closer to the MXene surface, providing defect-free membranes and preventing the formation of nonselective microvoids.

**Figure 4:**
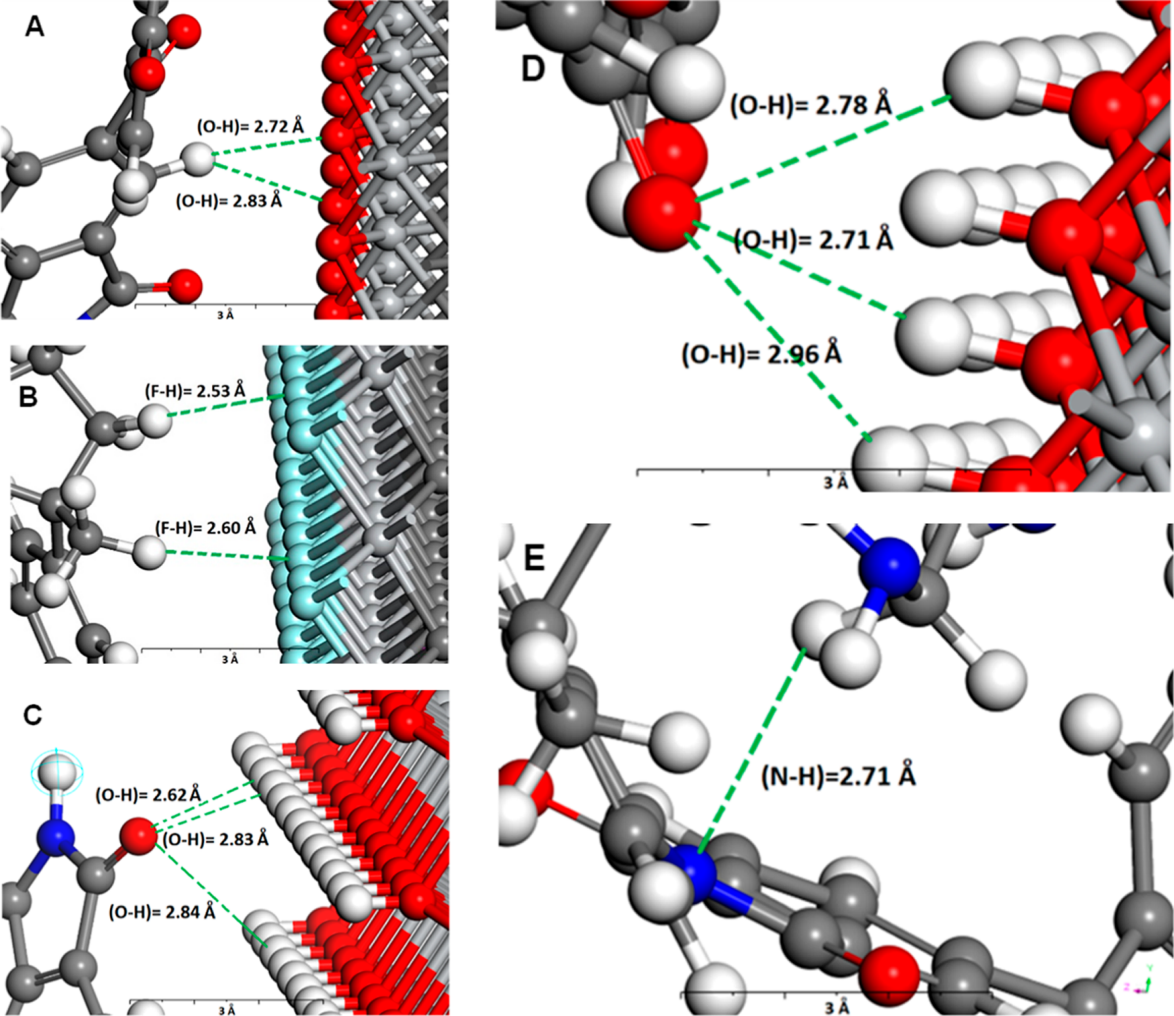
Hydrogen bonds formed from the interactions of Matrimid with (A) Ti_3_C_2_O_2_, (B) Ti_3_C_2_F_2_, (C) Ti_3_C_2_(OH)_2_, and (D,E) aminosilane-Ti_3_C_2_(OH)_2_. Light gray spheres = titanium atoms, dark gray = carbon, red = oxygen, white = hydrogen, dark blue = nitrogen, light blue = fluorine, and yellow = silicon.

Overall, the results demonstrated good compatibility between the Ti_3_C_2_T_*x*_ and Matrimid due to the possible hydrogen bonds that can form. The number of hydrogen bonds, especially strong and moderate hydrogen bonds (bond lengths of ≤3 Å), increases significantly when Ti_3_C_2_(OH)_2_ is functionalized with AEAPTMS. Furthermore, the presence of −NH_2_ in modified Ti_3_C_2_T_*x*_ also increased the number of hydrogen bonds in the range of 3–4 Å. This suggests that the surface modification enhances the interaction between Matrimid and MXene by increasing both the strength and the number of hydrogen bonds. These enhanced interactions play a critical role in improving the dispersion of the modified MXene and reducing the formation of nonselective microvoids within the membrane. Therefore, while −OH groups contribute to hydrogen bonding, the introduction of −NH_2_ groups through functionalization offers additional benefits, as evidenced by the simulation results. Figure 4 depicts several hydrogen bonds formed between the species in various samples.

### 3.3 Gas Separation Performance of Membranes

**Figure 5:**
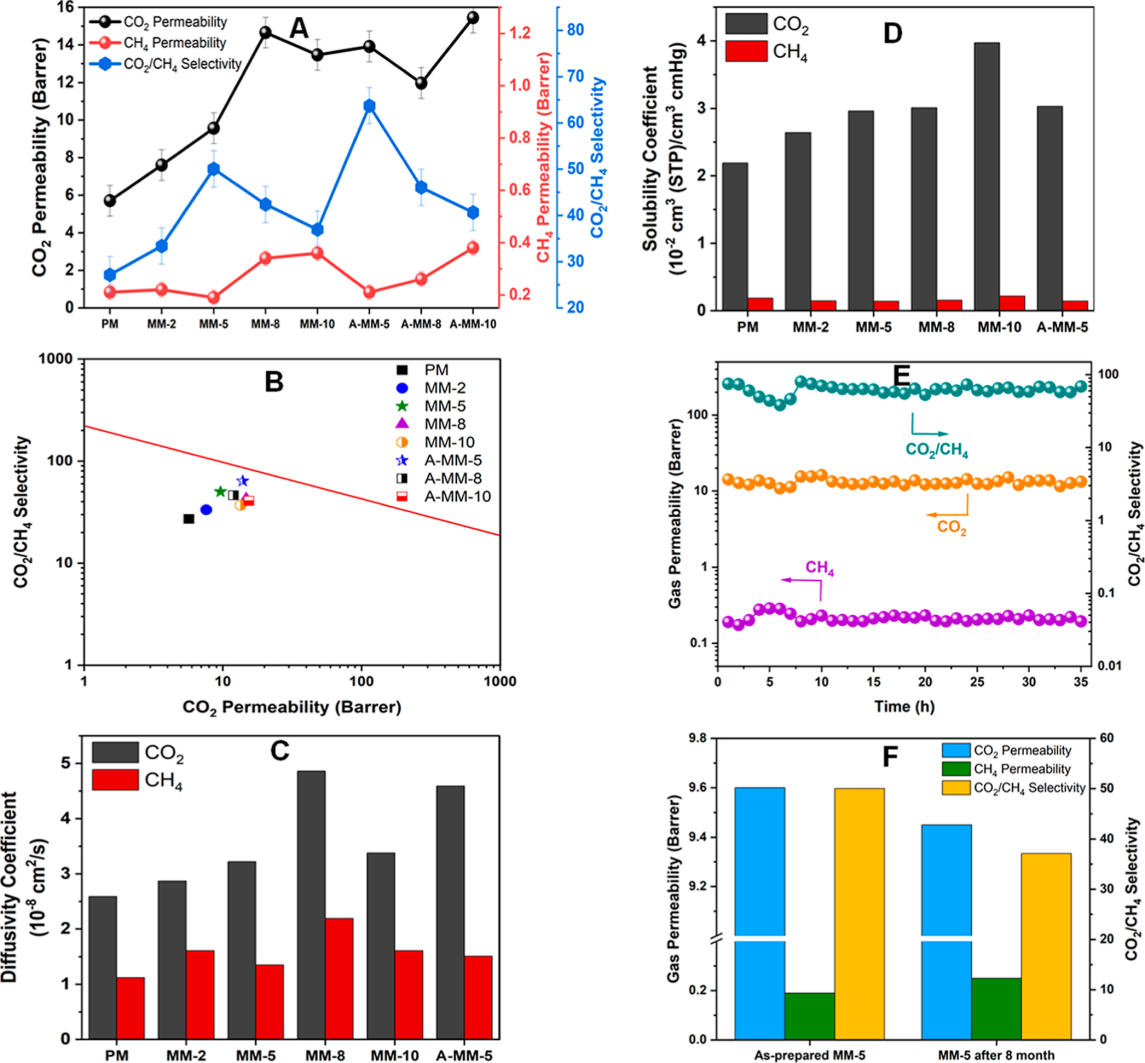
Gas transport properties of Ti_3_C_2_T_*x*_-Matrimid membranes. (A) CO_2_, CH_4_ permeabilities, and CO_2_/CH_4_ ideal selectivity. (B) Gas separation performance of the membranes developed in this study relative to the 2008 Robeson upper bound for CO_2_/CH_4_. (C) The diffusivity and (D) solubility coefficients of the pristine Matrimid and Ti_3_C_2_T_*x*_-Matrimid membranes at room temperature and 5 bar. (E) Long-term separation performance of A-MM-5 membrane at room temperature and 5 bar. (F) Comparison of the gas transport behavior of MM-5 membrane; before and after eight months.

The pure gas permeation of CO_2_ and CH_4_ through the membranes was measured at 25 °C and 5 bar, as shown in Figure 5A. The incorporation of 5 wt % pristine Ti_3_C_2_T_*x*_ in the Matrimid matrix (MM-5) substantially enhanced CO_2_ permeability to 9.56 Barrer (a 68% improvement compared to PM) and CO_2_/CH_4_ ideal selectivity to 50.04 (an 84% improvement compared to PM). Accordingly, the impact of Ti_3_C_2_T_*x*_ incorporation into the Matrimid matrix on  is presented in Figure S14, which shows an increase in . This significant improvement is attributed to improved interfacial interactions between pristine Ti_3_C_2_T_*x*_ and Matrimid chains, as confirmed by the characterization and simulation results. The lower CH_4_ permeability of MM-5 compared to those of PM and MM-2 can be attributed to the tortuosity of the diffusion path, resulting from the high aspect ratio of Ti_3_C_2_T_*x*_ MXenes, which hinders passage of CH_4_ molecules through the membrane.^53^ This phenomenon is commonly observed in MMMs.^54^ Moreover, increasing Ti_3_C_2_T_*x*_ MXenes loading to 8 and 10 wt % yielded a noticeable increase in CO_2_ permeability to 14.65 and 13.46 Barrer, respectively. However, this increase was accompanied by a decrease in CO_2_/CH_4_ ideal selectivity to 42.36 and 36.97, respectively. This trend can be ascribed to the formation of nonselective diffusive microvoids (Supporting Information, Figure S9C,D) due to the agglomeration of Ti_3_C_2_T_*x*_ MXenes within the polymer, which allows gas molecules to pass through the membranes with reduced selectivity. It is worth noting that water molecules intercalated between Ti_3_C_2_T_*x*_ layers have a dual effect on gas separation performance.^55−57^ Specifically, water molecules have been observed to facilitate the permeation of condensable CO_2_ molecules through the membrane, while acting as a barrier to CH_4_ transport, thereby impacting the overall separation efficiency. The introduction of Ti_3_C_2_T_*x*_ into Matrimid significantly enhances gas separation performance, surpassing the results reported for other fillers in Matrimid, as listed in Table S8 and shown in Figure S15. Notably, A-MM-5 exhibits a remarkable performance, positioning it as a highly efficient candidate for CO_2_/CH_4_ separation applications. Additionally, MMMs containing pristine Ti_3_C_2_T_*x*_ also demonstrate favorable permeability–selectivity trade-offs, further emphasizing the effectiveness of the Ti_3_C_2_T_*x*_ incorporation. This enhancement moves the separation performance of these Ti_3_C_2_T_*x*_-Matrimid membranes much closer to the 2008 Robeson upper (Figure 5B), representing a notable advancement over pristine Matrimid membranes.

To further assess the potential of Ti_3_C_2_T_*x*_ MXenes in improving the separation performance of MMMs, membranes composed of Matrimid and silane-agent-surface-functionalized Ti_3_C_2_T_*x*_ were tested for gas permeation. It was hypothesized that silane agents improve the interfacial interaction between functionalized MXene (A-Ti_3_C_2_T_*x*_) and polymer chains, influencing diffusivity. Additionally, they are believed to serve as CO_2_-philic carriers, which enhances solubility. Interestingly, by introduction of 5 wt % of A-Ti_3_C_2_T_*x*_ into Matrimid, CO_2_ permeability and CO_2_/CH_4_ ideal selectivity remarkably increased and reached to 13.91 Barrer and 63.73, respectively. This represents a 45.5% improvement in CO_2_ permeability and a 27.3% increase in CO_2_/CH_4_ ideal selectivity compared to the MM-5 membranes. By increasing the loading of A-Ti_3_C_2_T_*x*_ from 5 to 8 wt %, CO_2_ permeability decreased by approximately 13%, while CH_4_ permeability increased slightly. This behavior resulted in a reduction in overall CO_2_/CH_4_ selectivity. A high filler content often stiffens the polymer matrix, reducing chain mobility and free volume. As a result, CO_2_, which is more dependent on solubility and diffusivity, may experience a more significant reduction in transport compared to CH_4_, which is less affected by these factors. When 10 wt % of A-Ti_3_C_2_T_*x*_ was incorporated into Matrimid, increases in both CO_2_ and CH_4_ permeabilities and a reduction in CO_2_/CH_4_ selectivity were observed, indicating the agglomeration of A-Ti_3_C_2_T_*x*_ nanosheets. It should be noted that the membrane embedded with A-Ti_3_C_2_T_*x*_ has a better separation performance than the membranes embedded with pristine Ti_3_C_2_T_*x*_ at loadings of 8 and 10 wt %. This outcome highlights the significant impact of surface functionalization on gas separation performance. Molecular simulations further support these findings, revealing that the functionalized MXene (A-Ti_3_C_2_T_*x*_) exhibits stronger interactions with the polymer chains compared to the pristine MXene. These stronger interactions are primarily attributed to the formation of stronger hydrogen bonds between the functional groups on the A-Ti_3_C_2_T_*x*_ surface and the polymer chains. This stronger hydrogen bonding leads to a more cohesive and stable interface, promoting better dispersion of MXene nanosheets within the polymer matrix and preventing the formation of nonselective microvoids.

The enhancements observed in CO_2_ permeabilities correlate with improvement in both diffusivity and solubility coefficients within Ti_3_C_2_T_*x*_-Matrimid at different loadings. To better understand the transport mechanism, the diffusivity and solubility coefficients were calculated using the time-lag method with results displayed in Figure 5C,D. The diffusivity coefficient of CO_2_ (2.59 × 10^–8^ cm^2^/s) in the PM membrane is higher than CH_4_ (1.12 × 10^–8^ cm^2^/s) because of CO_2_’s lower kinetic diameter (3.3 Å) compared to CH_4_ (3.8 Å). Introducing Ti_3_C_2_T_*x*_ MXene into Matrimid led to increased diffusivity coefficients for both CO_2_ and CH_4_ molecules, which can be attributed to factors such as the presence of interlayer nanogalleries in Ti_3_C_2_T_*x*_ and the disruption of Matrimid chains. These factors create cavities within the membrane structure that facilitate the rapid transport of CO_2_ molecules.^20^ Moreover, MD simulations by Shamsabadi et al. confirmed that the presence of Ti_3_C_2_T_*x*_ MXene increased the fractional free volume (FFV), providing further explanation for the observed rise in diffusivity coefficients.^58^ CO_2_ molecules have a higher solubility coefficient (2.41 × 10^–2^ cm^3^ (STP)/cm^3^ cmHg) than CH_4_ (0.205 × 10^–2^ cm^3^ (STP)/cm^3^ cmHg) in PM membrane due to their greater condensability, resulting from a higher critical temperature. The quadrupole moments of CO_2_ molecules create a strong affinity for the carbonyl groups present in the Matrimid structure, making them more soluble.^59^ Consequently, the CO_2_ solubility coefficients increased in MMMs with the incorporation of pristine Ti_3_C_2_T_*x*_ MXenes, which exhibit a higher affinity toward CO_2_ compared to CH_4_. For example, integrating 5 wt % of pristine Ti_3_C_2_T_*x*_ into Matrimid increased CO_2_ solubility coefficient to 3.26 (×10^–2^ cm^3^ (STP)/cm^3^ cmHg). This phenomenon has also been observed elsewhere, where CO_2_ was detected within an N_2_ atmosphere using a MXene sensor, further confirmed through first-principles density functional theory.^60^ Additionally, polymer disruption allows gas molecules to penetrate the free volume around the polymer chains at the Ti_3_C_2_T_*x*_–Matrimid interface, indicating the availability of additional sites for gas molecule adsorption within the MMMs.^58^ The enhanced permeability of the A-MM-5 membrane compared to the MM-5 membrane is primarily due to a slight increase in the CO_2_ solubility coefficient (∼3%) and a significant rise in the diffusivity coefficient (∼35%). This improvement is attributed to the stronger interfacial interaction between A-Ti_3_C_2_T_*x*_ and Matrimid chains, as confirmed by molecular simulations, leading to more uniform dispersion and minimal nonselective voids within the membrane structure. These findings align with the hypothesis that silane agents enhance interfacial interactions and diffusivity coefficient, while the modest increase in CO_2_ solubility coefficient also contributes to the overall improvement in permeability.

**Figure 6:**
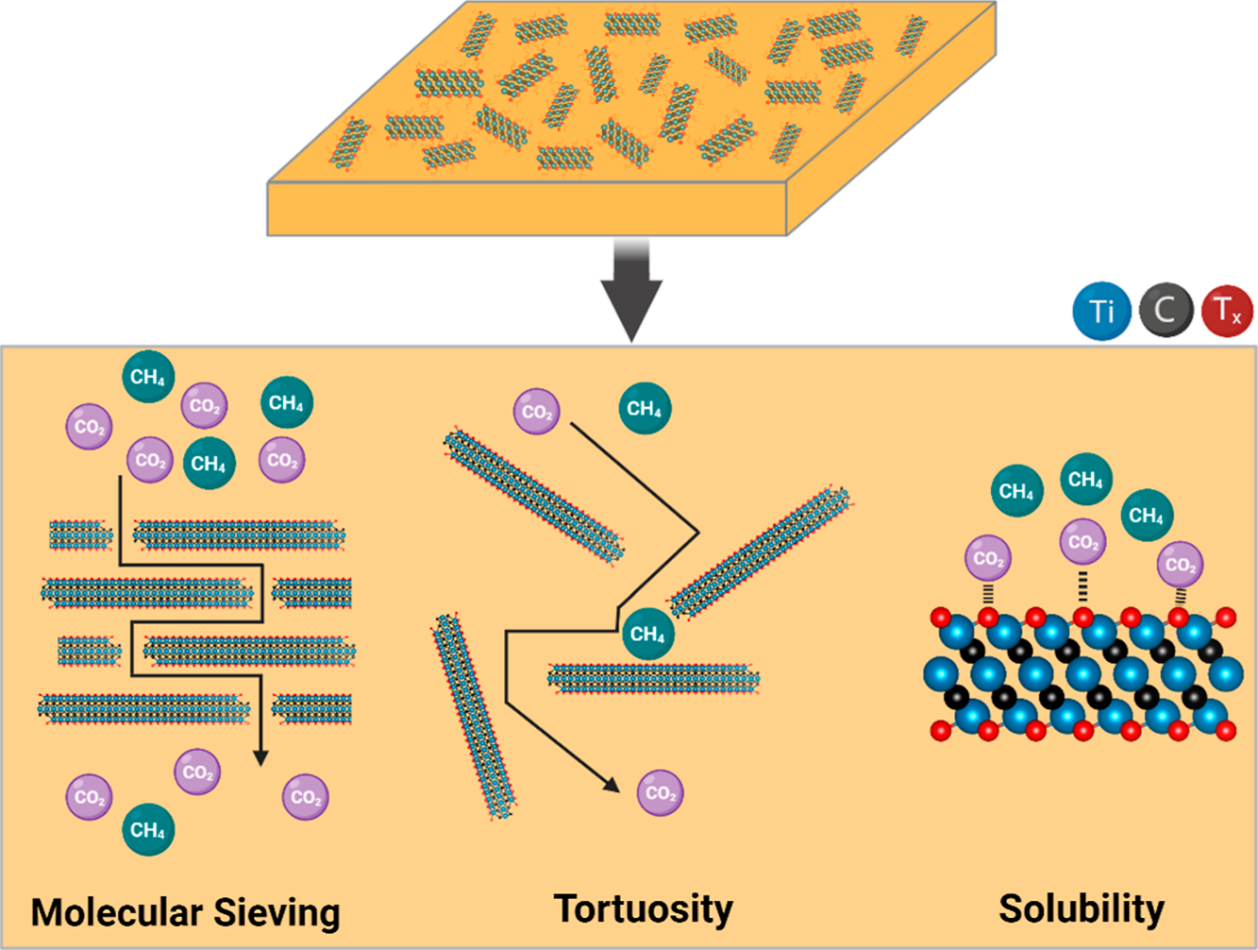
Proposed transport mechanisms of CO_2_ and CH_4_ gas molecules through the Ti_3_C_2_T_*x*_/Matrimid and A-Ti_3_C_2_T_*x*_/Matrimid MMMs.

Figure 5E demonstrates excellent separation performance of the A-MM-5 membrane for CO_2_ and CH_4_ during a 35 h continuous permeation test (results for the MM-5 membrane are shown in Figure S16). The time-invariant CO_2_ and CH_4_ permeabilities and CO_2_/CH_4_ ideal selectivity indicate the structural stability of the A-MM-5 and MM-5 membranes, which is crucial for industrial applications. Furthermore, the high separation performance of the MM-5 membrane is well maintained even after 8 months of storage, as depicted in Figure 5F. This longevity suggests the membrane’s excellent chemical stability, further affirmed by the Matrimid chains effectively shielding the MXene surface and preventing oxidation of Ti_3_C_2_T_*x*_ nanoflakes. Figure 6 schematically illustrates the transport pathways of CO_2_ and CH_4_ through the prepared MMMs to provide a deeper understanding of the separation mechanism. The incorporation of pristine Ti_3_C_2_T_*x*_ or A-Ti_3_C_2_T_*x*_ nanosheets significantly influences the governing separation processes. While surface functionalization is not expected to alter the separation mechanism, this study highlights the synergistic effects of molecular sieving and solution-diffusion mechanisms, which together enhance both CO_2_/CH_4_ selectivity and CO_2_ permeability. First, the interlayer nanogalleries formed by MXene nanosheets within the Matrimid matrix (with an interlayer spacing of approximately 0.35 nm) enable molecular sieving. CO_2_ that has a smaller kinetic diameter can easily diffuse through these interlayer spaces, while CH_4_ that has a larger kinetic diameter cannot. Second, the inclusion of pristine and functionalized Ti_3_C_2_T_*x*_ nanosheets creates an extended diffusion pathway, increasing the diffusivity selectivity for CO_2_/CH_4_. Lastly, the strong affinity between CO_2_ molecules and the surface of Ti_3_C_2_T_*x*_ nanosheets enhances solubility selectivity. This preferential adsorption of CO_2_, driven by its higher quadrupole moment compared to CH_4_, results in a substantial improvement over the performance of the pristine Matrimid membrane.

## 4 Conclusions

This work demonstrated the successful integration of pristine and surface-modified Ti_3_C_2_T_*x*_ MXenes into Matrimid 5218 polymer to create mixed matrix membranes (MMMs) with enhanced CO_2_/CH_4_ gas separation performance. MXene–Matrimid membranes showed over 2-fold enhancement in both CO_2_ permeability and CO_2_/CH_4_ ideal selectivity. These improvements can be attributed to the formation of strong interfacial interactions between Matrimid chains and the MXenes, corroborated by molecular simulations and characterization results. The uniform dispersion of the MXenes within the membrane matrix created favorable channels for fast CO_2_ transport across the membranes leading to enhanced CO_2_ permeabilities. Moreover, the increased CO_2_/CH_4_ ideal selectivities can be ascribed to more tortuosity and longer gas transport pathways created by the MXene nanosheets. The silane-agent-surface-functionalized Ti_3_C_2_T_*x*_ at 5 wt % loading, further improved gas separation performance by enhancing both CO_2_ permeability (45.5%) and CO_2_/CH_4_ ideal selectivity (27.3%) compared to the 5 wt % pristine Ti_3_C_2_T_*x*_. This improvement is due to stronger interfacial interactions and CO_2_-philic properties. These findings highlight the crucial role of surface modification in optimizing MXene-based MMMs. The inclusion of either the pristine Ti_3_C_2_T_*x*_ or functionalized Ti_3_C_2_T_*x*_ enhances both diffusivity and the solubility coefficients of condensable CO_2_ molecules due to their better affinity to the polymer. Long-term and aging experiments showed the outstanding mechanical and chemical stability of the MMMs. While the performance of the pristine Matrimid membranes are well below the 2008 Robeson upper bound, the performances of the corresponding MMMs are substantially higher and are close to the upper bound. These findings provide valuable insights for designing MMMs with superior gas separation performance, contributing to the advancement of more efficient gas separation technologies.

## Supporting Information Available

Molecular simulation details; schematic of gas separation setup; additional characterization results, including SEM, AFM, XPS, Raman, DLS, and TGA, presented in the form of Figures and Tables (PDF)
