# Supplementary material for: Substantially Improving CO2 Permeability and CO2/CH4 Selectivity of Matrimid Using Functionalized-Ti3C2Tx
Source: ACS Appl Mater Interfaces. 2025 Jan 2;17(2):3897–910. doi: 10.1021/acsami.4c17315 (PMC11744502; doi:10.1021/acsami.4c17315)
Supplement: Supplementary file 1 — am4c17315_si_001.pdf [file am4c17315_si_001.pdf]

Supporting Information (SI)

Substantially Improving CO_2 Permeability and CO_2/CH_4 Selectivity of Matrimid Using Functionalized-$Ti_3C_2T_x$

Mohammad Mozafari[1], Saeed Khoshhal Salestan[2,3], Ahmad Arabi Shamsabadi[1,4], Kritika Jha[4], Manushree Tanwar[4], Hyehyun Kim[5], Zahra Fakhraai[4], and Masoud Soroush[1,5,*]

[1] Department of Chemical and Biological Engineering, Drexel University, Philadelphia, PA 19104, USA

[2] Department of Chemical Engineering, Babol Noshirvani University of Technology, Babol 47148-71167, Iran

[3] Department of Mechanical Engineering, 10-367 Donadeo Innovation Center for Engineering, Advanced Water Research Lab (AWRL), University of Alberta, Edmonton, Alberta T6G 1H9, Canada

[4] Department of Chemistry, University of Pennsylvania, Philadelphia, PA, 19104, USA

[5] Department of Materials Science and Engineering, Drexel University, Philadelphia, PA 19104, USA

Submitted for publication in *ACS Applied Materials & Interfaces*

December 25, 2024

Keywords: Mixed-matrix membrane, $Ti_3C_2T_x$ MXene; surface functionalization; Matrimid; gas separation.

* Corresponding author: soroushm@drexel.edu

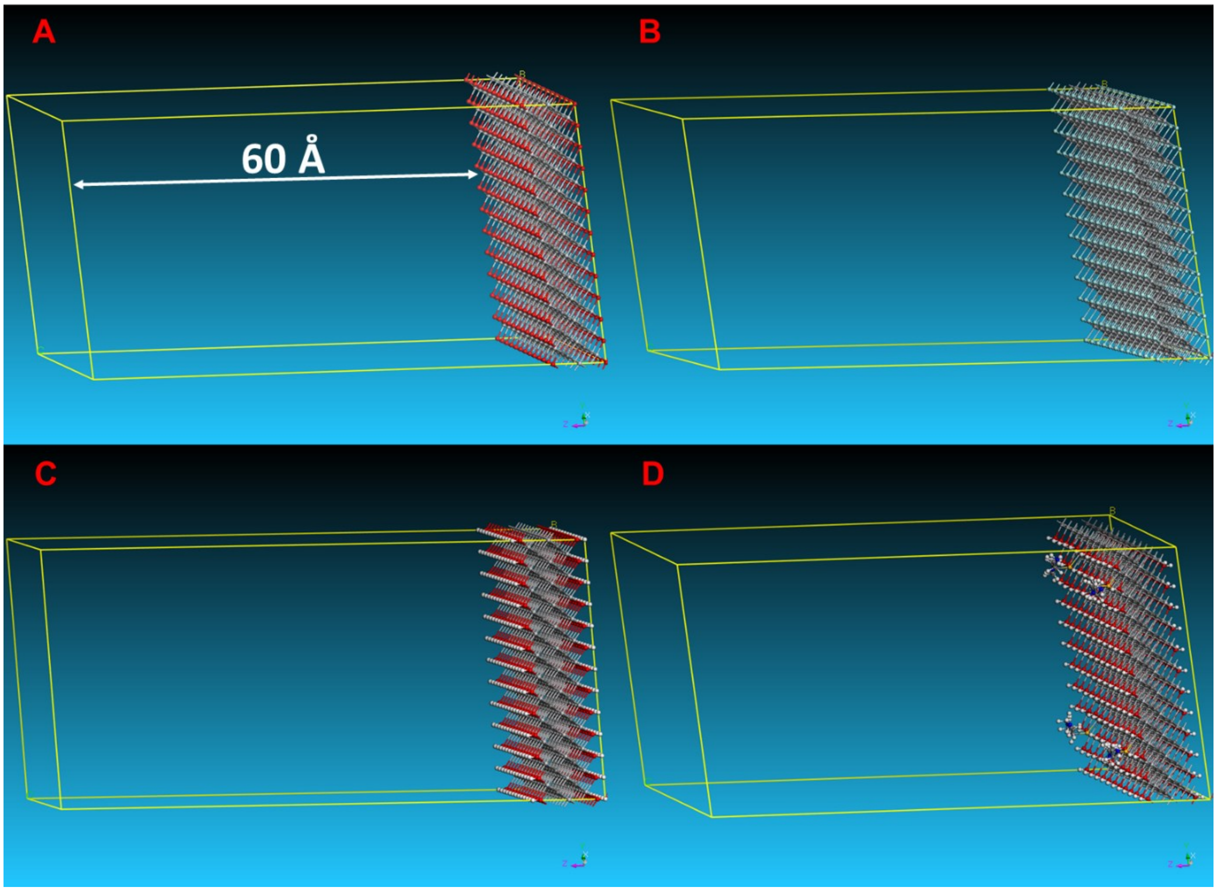

Figure S1. Vacuum slabs of $Ti_3C_2T_x$ MXene with different functional groups obtained using molecular simulations; (A) $Ti_3C_2O_2$, (B) $Ti_3C_2F_2$, (C) $Ti_3C_2(OH)_2$, and (D) aminosilane-$Ti_3C_2(OH)_2$. Light gray spheres = titanium, dark gray = carbon, red = oxygen, white = hydrogen, dark blue = nitrogen, light blue = fluorine, and yellow = silicon.

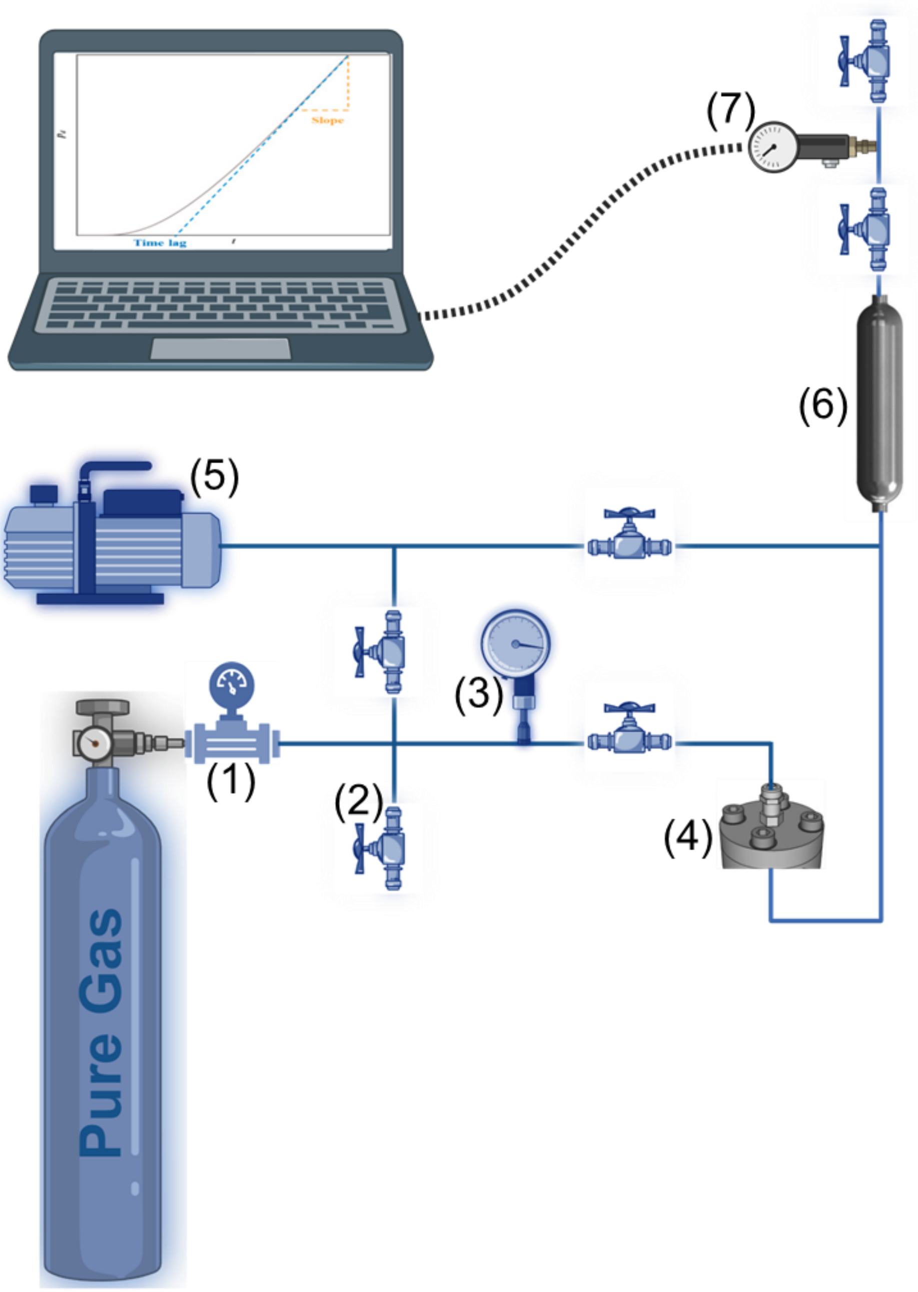

Figure S2. Schematic representation of the constant volume/variable pressure gas separation set-up. (1) Pressure regulator, (2) diaphragm-sealed valve, (3) digital pressure gauge, (4) membrane holder, (5) vacuum pump, (6) gas sample cylinder, and (7) pressure data logger (Track-It^{TM} Vacuum/Temperature data logger, 760 to 0 Torr).

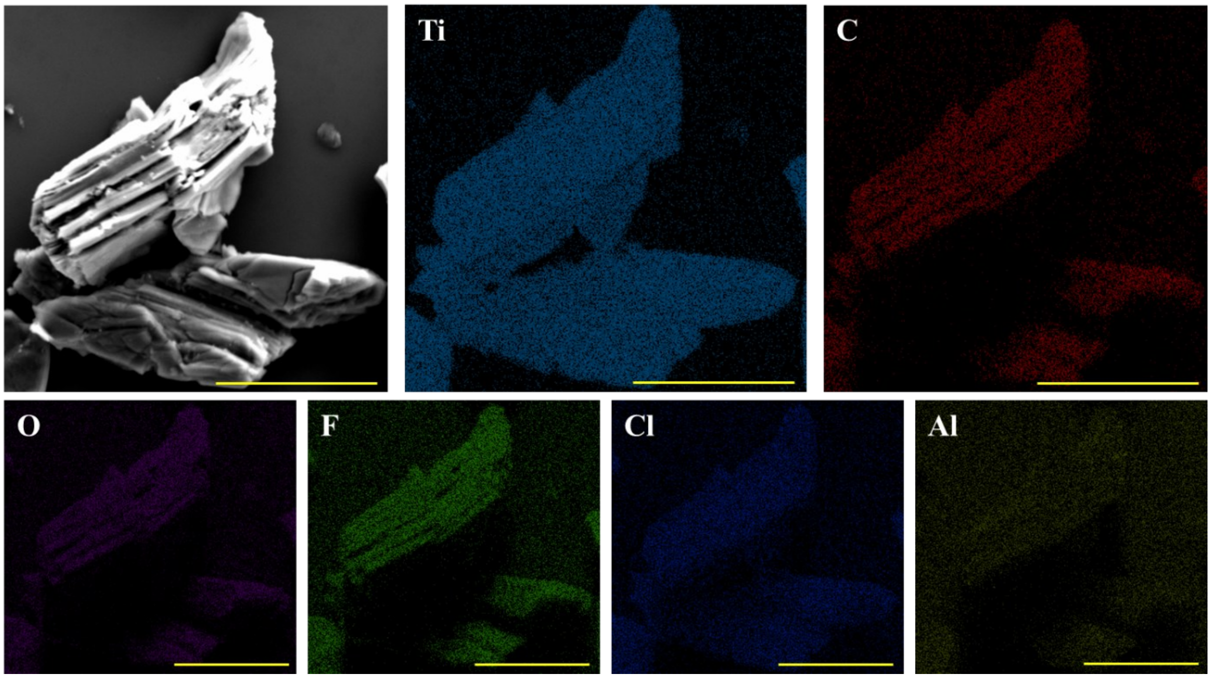

Figure S3. SEM image and corresponding energy dispersive X-ray spectroscopy (EDS) mapping of multilayer $Ti_3C_2T_x$ MXene. Scale bar: 10 μm.

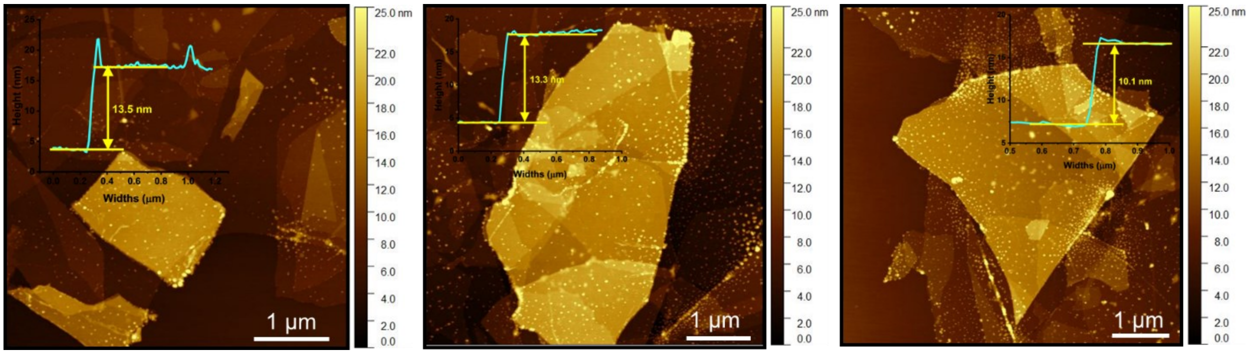

Figure S4. AFM images of randomly stacked $Ti_3C_2T_x$ nanosheets on a Si substrate. The inset shows the height profile of the corresponding $Ti_3C_2T_x$ nanosheets.

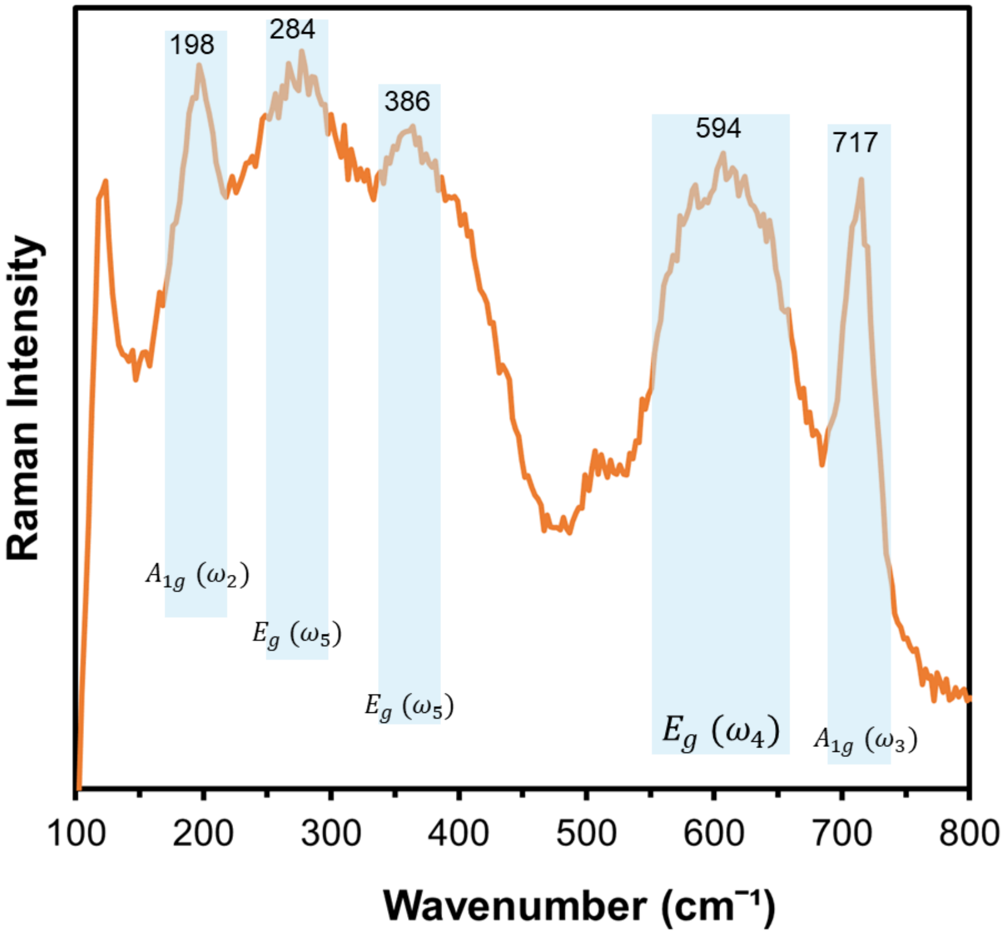

Figure S5. Raman spectrum of pristine $Ti_3C_2T_x$.

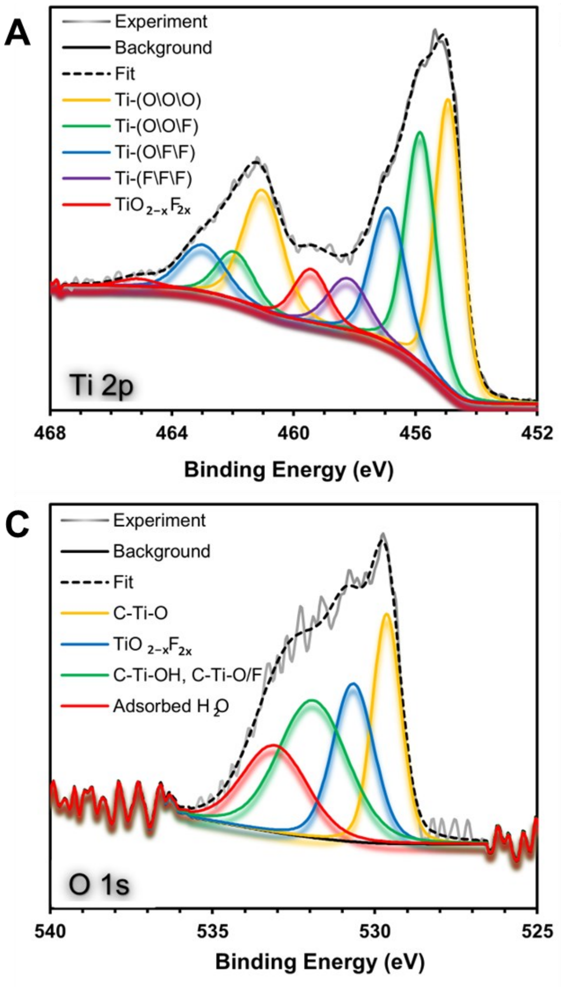

Figure S6. High-resolution XPS spectra of $Ti_3C_2T_x$ MXene for (A) Ti 2p, (B) C 1s, (C) O 1s, and (D) F 1s.

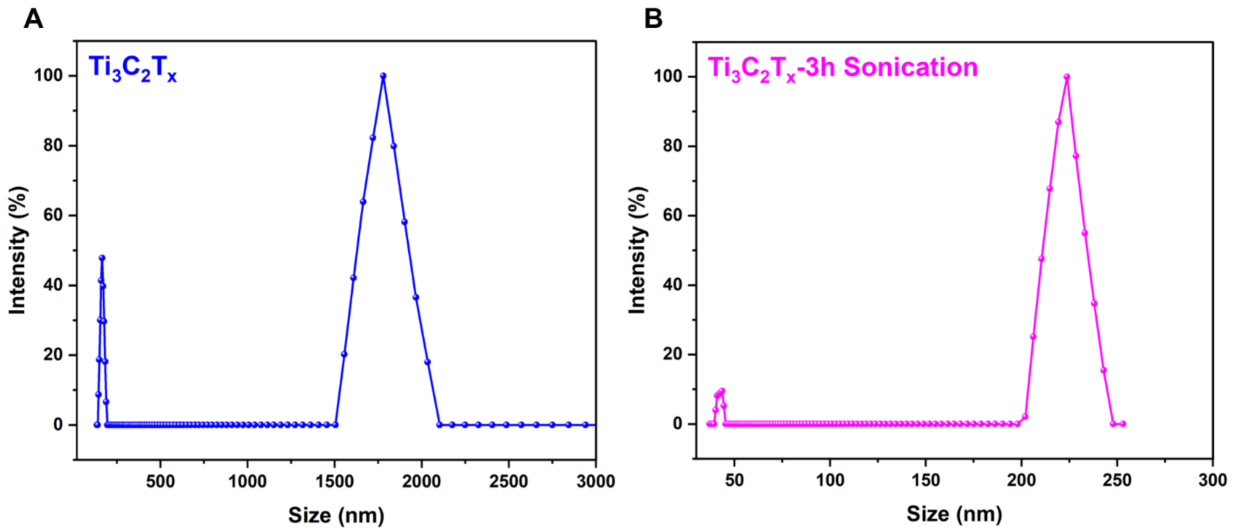

Figure S7. Intensity particle-size distribution for pristine $Ti_3C_2T_x$ (A) before and (B) after 3 hours of sonication.

Supporting Note 1:

In this study, bath sonication with a sonic power of 55 (W) was employed for 3 h to reduce the flake size of as-synthesized $Ti_3C_2T_x$ MXenes. Previous research has indicated that the crystallinity of $Ti_3C_2T_x$ can be evaluated by the presence of the (002) peak in X-ray diffraction (XRD) patterns[1]. XRD measurements of $Ti_3C_2T_x$ films, prepared via vacuum-assisted filtration from colloidal solutions, showed that the (002) peak positions remained around 6.8°, suggesting consistent interlayer spacing despite the size reduction of flakes induced by sonication. Raman spectroscopy analysis reveals that the A 1g (C) and A 1g (Ti, C, O) modes slightly shift to lower wavenumbers with extended sonication[2]. These slight shifts are attributed to a higher ratio of flake edges to basal planes, consistent with an increase in defect concentration.

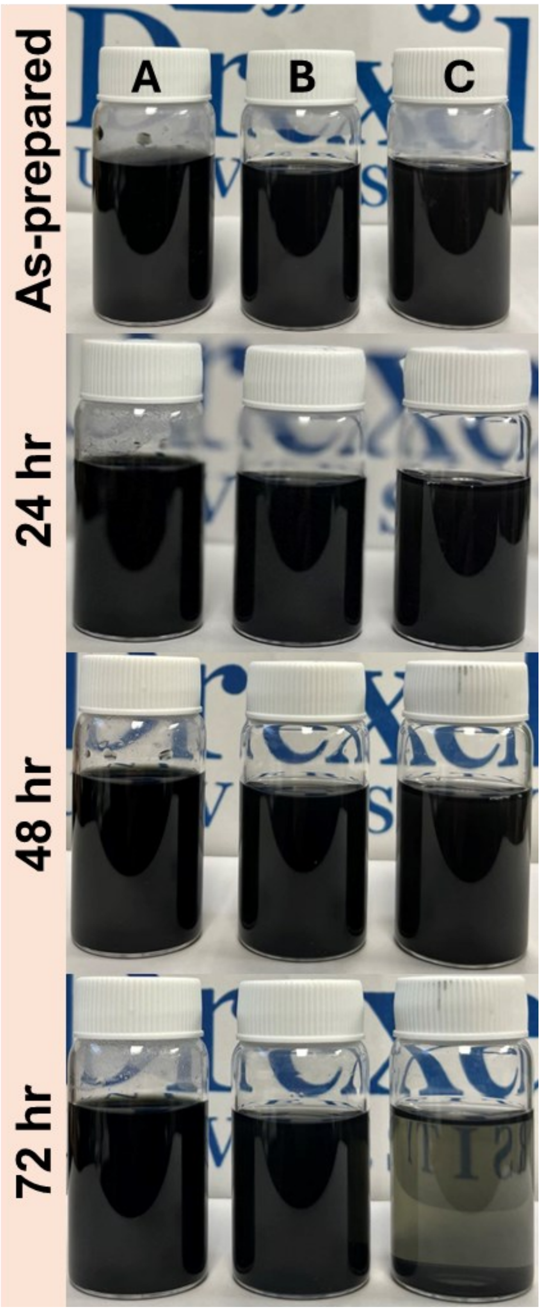

Figure S8. Optical images showing the dispersions of $Ti_3C_2T_x$ in deionized water (column A), $Ti_3C_2T_x$ in DMF (column B), and A-$Ti_3C_2T_x$ in DMF (column C) at different time points.

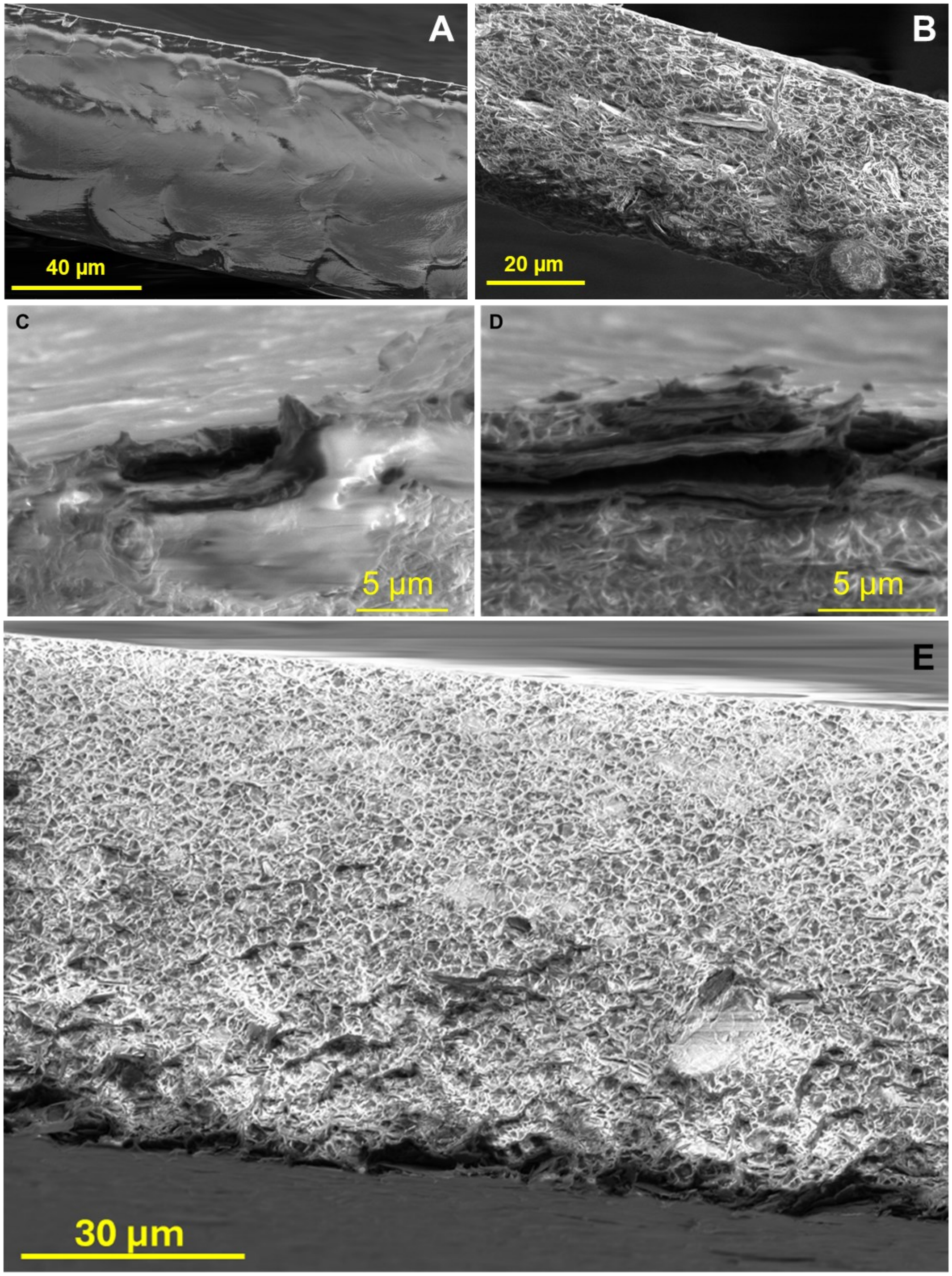

Figure S9. Cross–sectional SEM images of (A) PM and (B) MM-5, (C) MM-8, (D) MM-10, and (E) A-MM-5 membranes.

Supporting Note 2:

In the FTIR spectrum of pristine Matrimid, the absorption bands at 714 and 1295 cm^{-1} correspond to the stretching modes of C–N–C in the imide group, while the band at 1363 cm^{-1} indicates the stretching vibration of N–H bonds in secondary amines. The peaks at 1775 and 1667 cm^{-1} signify the symmetric stretching of C=O groups in imide and C=O stretching modes in benzophenone, respectively. The stretching vibrations of the C=C aromatic bonds are observed at 1506 and 1614 cm^{-1}. Additionally, the absorption peak at 2960 cm^{-1} can be attributed to C–H stretching in the methyl group[3, 4].

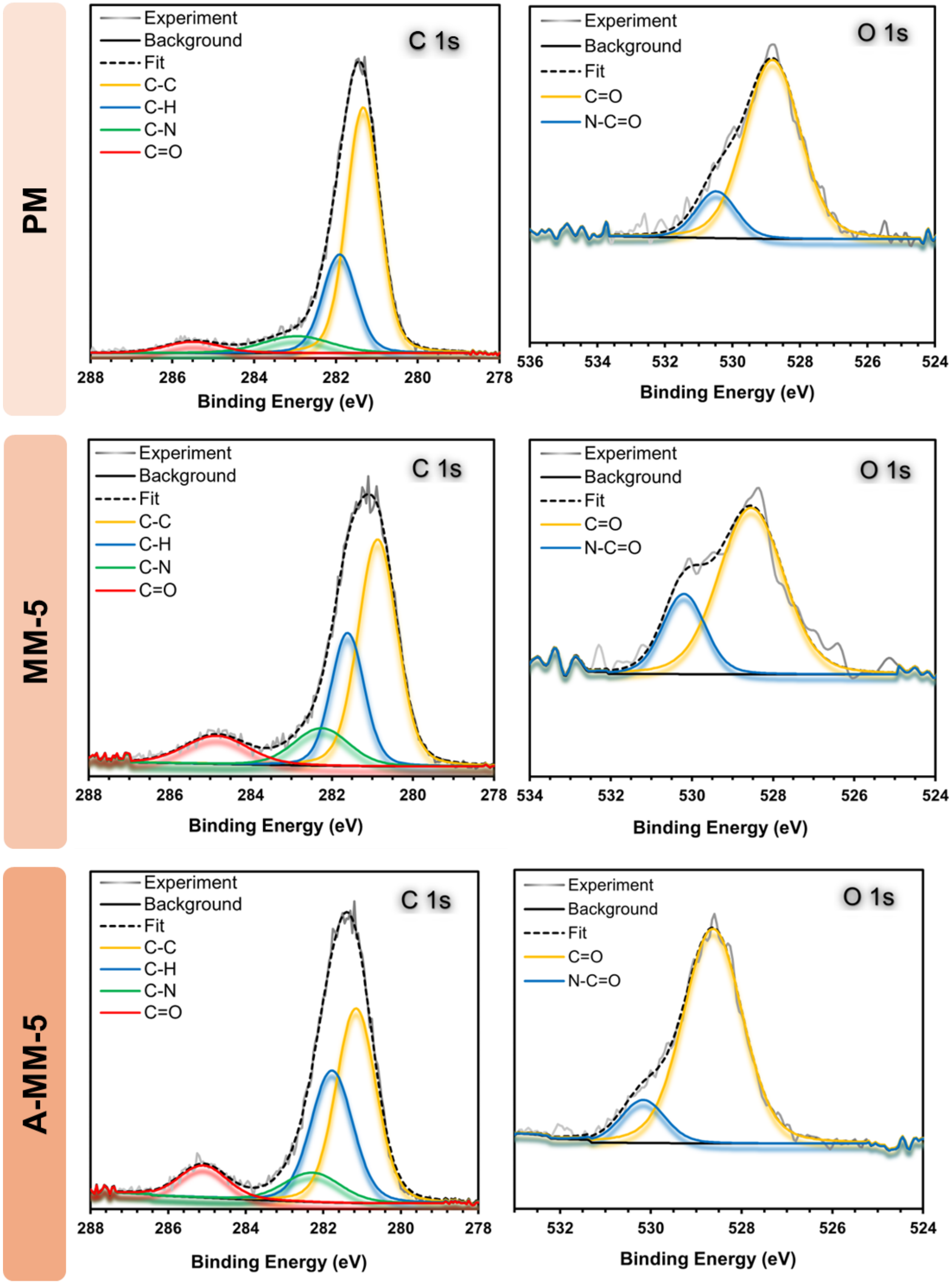

Figure S10. High-resolution C 1s and O 1s XPS spectra of the PM, MM-5 and A-MM-5 membranes.

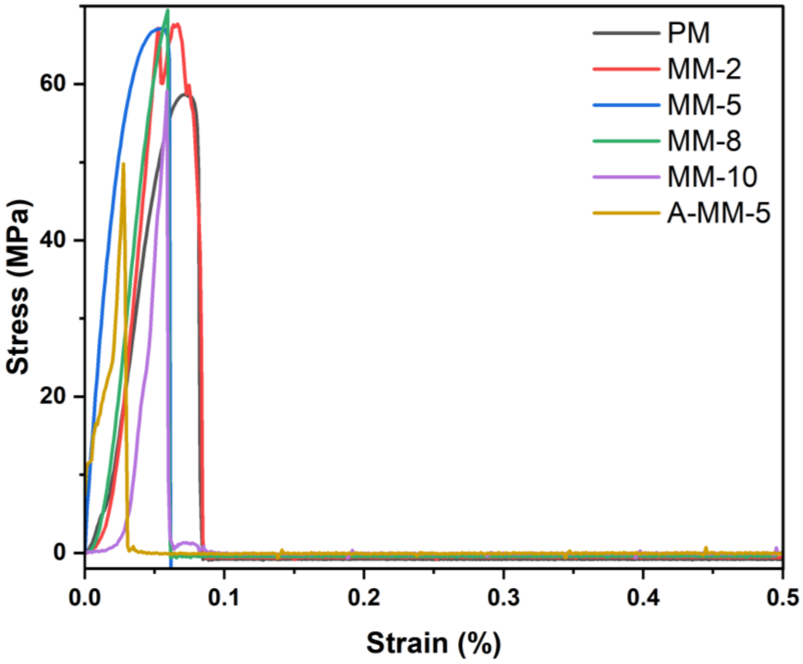

Figure S11. Tensile stress-strain curves of the pristine Matrimid and MMMs, highlighting the mechanical behavior and tensile properties of the materials.

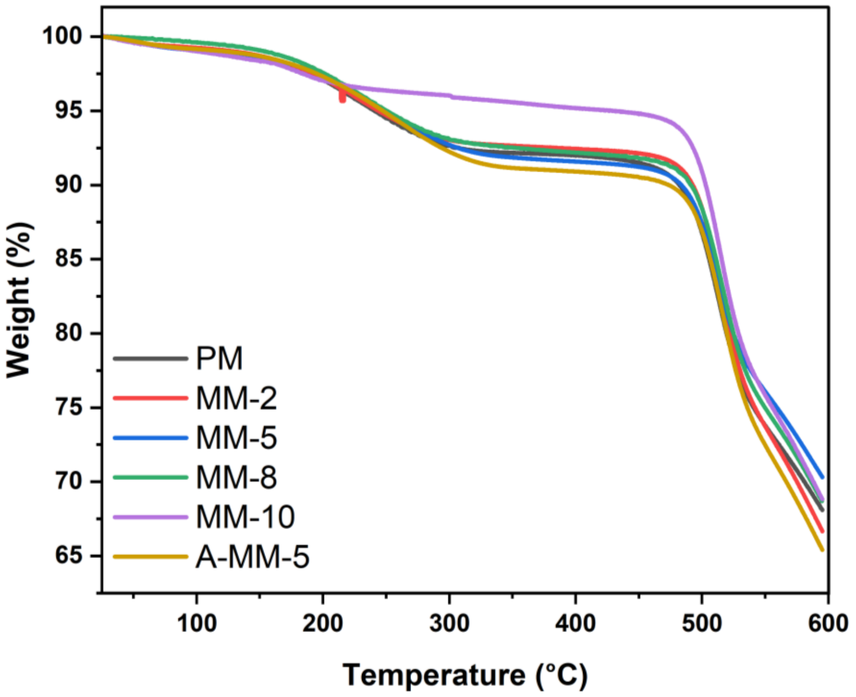

Figure S12. TGA thermograms of the pristine Matrimid and MMMs.

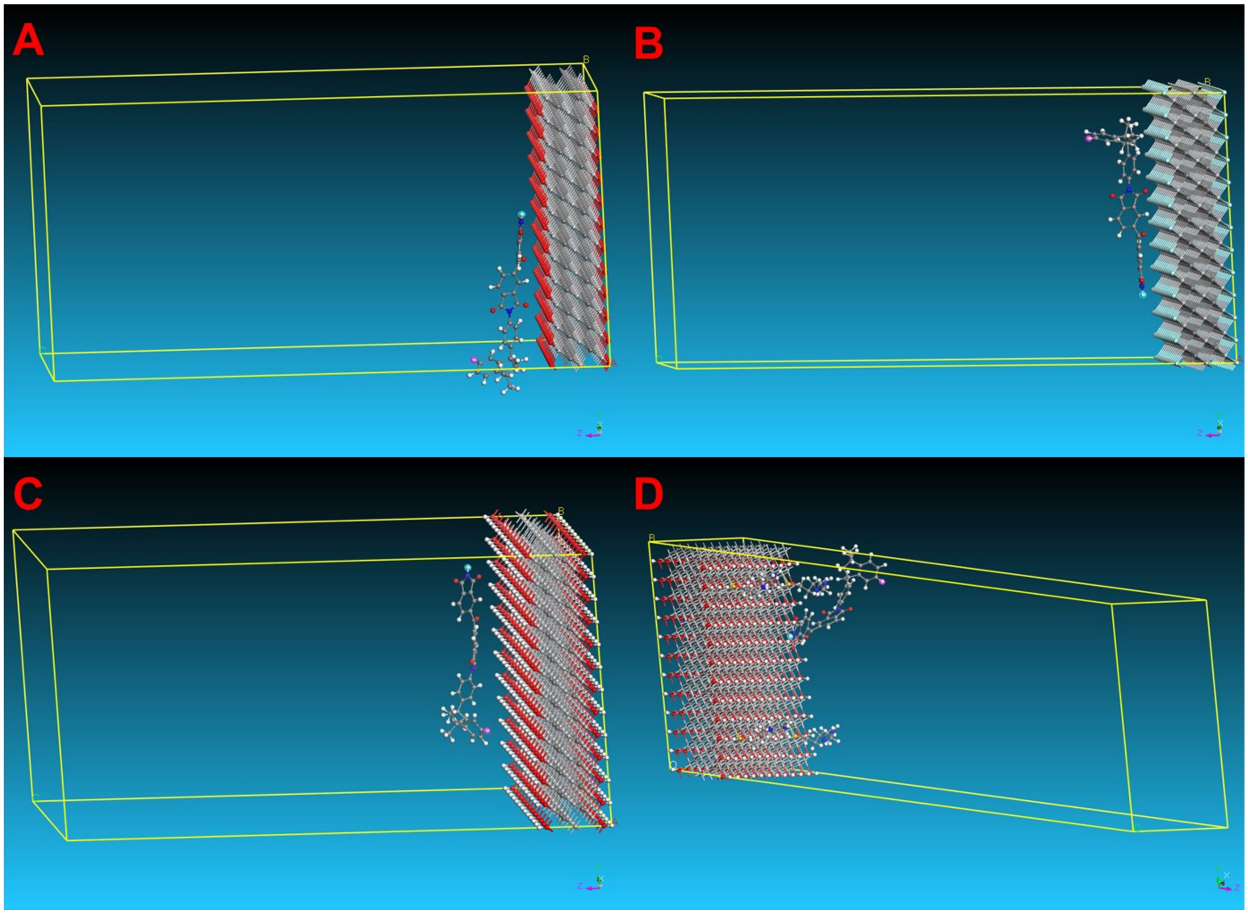

Figure S13. The preferential configurations of Matrimid's repeating unit interacting with the surface functional groups of the MXene obtained using molecular simulations; (A) $Ti_3C_2O_2$, (B) $Ti_3C_2F_2$, (C) $Ti_3C_2(OH)_2$, and (D) aminosilane-$Ti_3C_2(OH)_2$. Light gray spheres = titanium, dark gray = carbon, red = oxygen, white = hydrogen, dark blue = nitrogen, light blue = fluorine, and yellow = silicon.

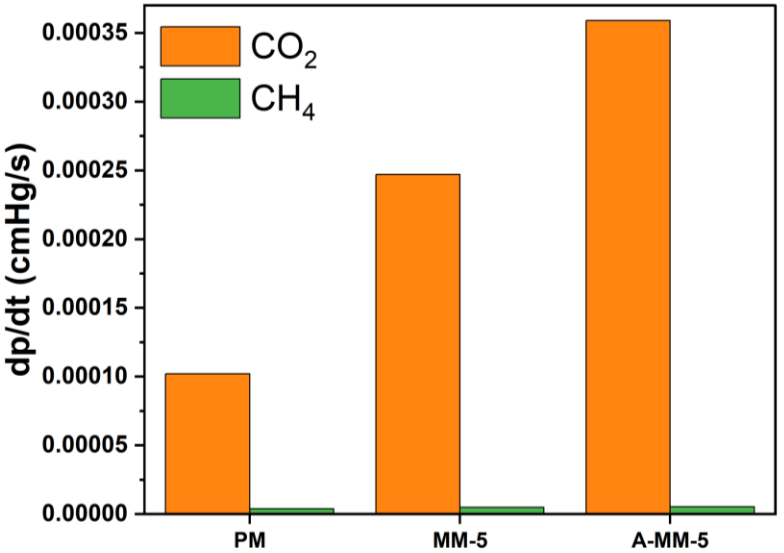

Figure S14. The rate of change of the permeate pressure (cmHg) when the pressure changes linearly with time (s).

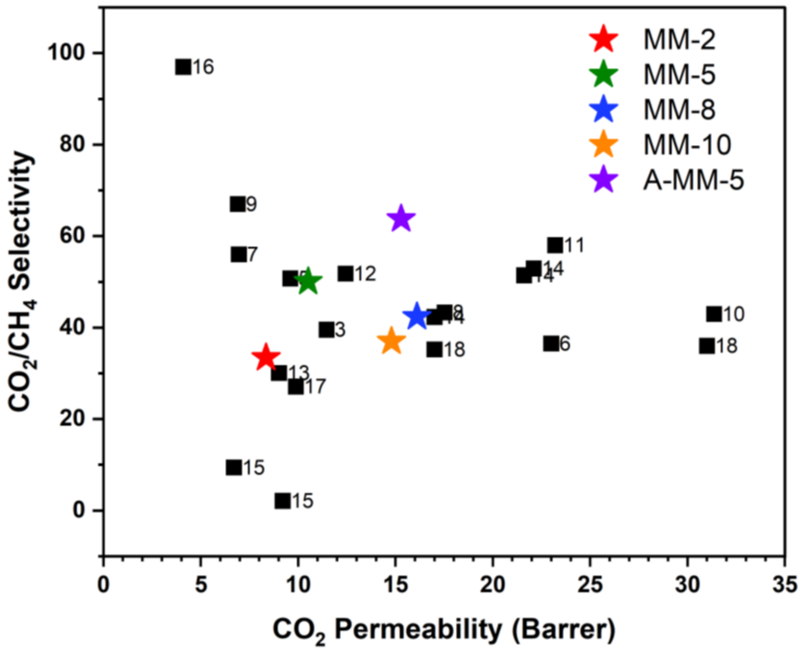

Figure S15. Comparison of the performances of the Matrimid-based MMMs with those of membranes reported in the literature (numbers next to the symbols indicate the references where the results reported).

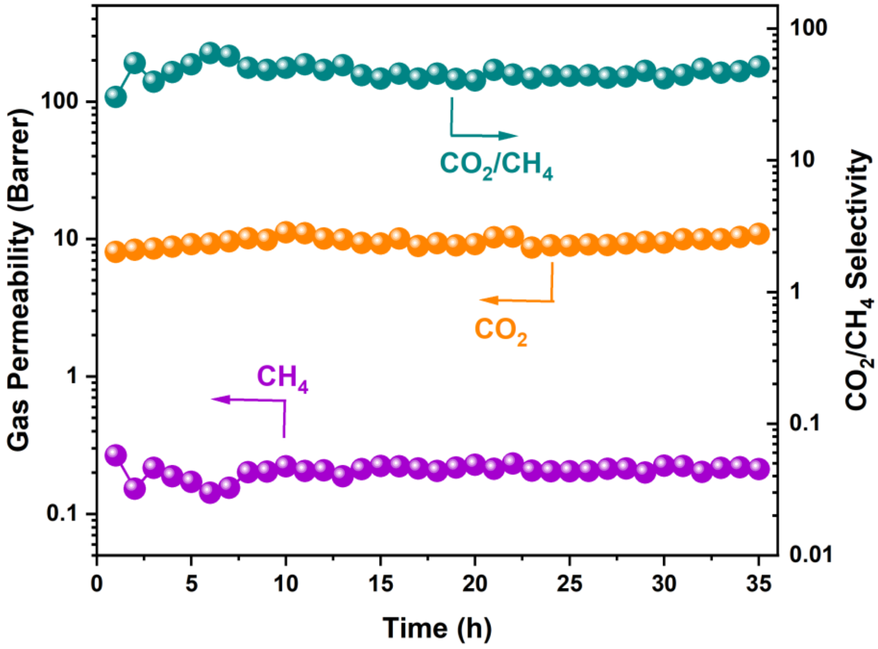

Figure S16. Long-term separation performance of MM-5 membrane at room temperature and 5 bar.

Table S1. Casting solution compositions of the prepared membranes.

Matrimid (wt.%)	MXene (wt.%)	DMF solvent (wt.%)	Membrane	Name
10	0	90	Pristine Matrimid	PM
9.8	0.2	90	Matrimid-2 wt.% $Ti_3C_2T_x$	MM-2
9.5	0.5	90	Matrimid-5 wt.% $Ti_3C_2T_x$	MM-5
9.2	0.8	90	Matrimid-8 wt.% $Ti_3C_2T_x$	MM-8
9	1	90	Matrimid-10 wt.% $Ti_3C_2T_x$	MM-10
9.5	0.5	90	Matrimid-5 wt.% AEAPTMS-functionalized $Ti_3C_2T_x$	A-MM-5

Table S2. Unit cell parameters of different $Ti_3C_2T_2$ samples with different functionalities calculated by molecular simulation.

MXene formula	Lattice parameters (Å)		Atom position	
$Ti_3C_2O_2$	a	3.039	Ti1	1b (0,0,0.5)
	c	18.833	Ti2	2d (1/3,2/3,0.6363)
	d	6.942	C	2d (1/3,2/3,0.4334)
			O	2c (0,0,0.3578)
$Ti_3C_2F_2$	a	3.076	Ti1	1b (0,0,0.5)
	c	25.3558	Ti2	2d (1/3,2/3,0.5928)
	d	7.212	C	2d (1/3,2/3,0.4492)
			F	2c (0,0,0.3578)
$Ti_3C_2(OH)_2$	a	3.086	Ti1	1b (0,0,0.5)
	c	18.5662	Ti2	2d (1/3,2/3,0.6269)
	d	9.220	C	2d (1/3,2/3,0.4310)
			O	2c (0,0,0.3044)
			H	2c (0,0,0.2517)

Table S3. XPS peak-fitting results for $Ti_3C_2T_x$.

**Region**	**BE (eV)**	**FWHM (eV)**	**Assigned to**
Ti $2p_{3/2}$ ($2p_{1/2}$)	454.9 (461.1)	1.1 (1.5)	Ti-(O\O\O)
	455.8 (461.9)	1.1 (1.5)	Ti-(O\O\F)
	456.8 (462.9)	1.1 (1.5)	Ti-(O\F\F)
	458.2 (464.3)	1.1 (1.5)	Ti-(F\F\F)
	459.4 (465.1)	1.3 (1.4)	$TiO_{2-x}F_{2x}$
C 1s	281.8	1.0	C-Ti
	284.7	1.8	C-C
	286.0	2.1	C-O
	288.7	2.2	O-C=O
O 1s	529.6	1.0	C-Ti-O
	530.6	1.5	$TiO_{2-x}F_{2x}$
	531.9	2.5	C-Ti-OH, C-Ti-O/F
	533.0	2.3	Adsorbed H_2O
F 1s	685.0	1.4	C-Ti-F
	686.7	1.1	F Contamination

Table S4. Raman peaks for the $Ti_3C_2T_x$ and MM-5 membrane.

Peak	Ti-C	H atoms	O atoms (E_g)	H atoms	O atoms (A_{1g})	C atoms (E_g)	C–C (A_{1g})
$Ti_3C_2T_x$	193	279	361	504	577	611	713
MM-5	196	284	368	508	584	625	721

Table S5. XPS peak-fitting results (C 1s and O 1s) for the pristine Matrimid, MM-5, and A-MM-5 membranes.

**Region**	**Functional groups**	**Pristine Matrimid (eV)**	**MM-5 (eV)**	**A-MM-5 (eV)**
C 1s	C–C	281.3	280.9	281.1
	C–H	281.9	281.6	281.7
	C–N	282.9	282.3	282.2
	C=O	285.4	284.9	285.1
O 1s	C=O	528.8	528.5	528.6
	N–C=O	530.5	530.2	530.1

Table S6. Glass transition temperature (T_g) of the pristine Matrimid and MMMs.

**Membrane**	**Glass Transition Temperature (°C)**
PM	318
MM-2	321
MM-5	325
MM-8	322
MM-10	324
A-MM-5	323

Table S7. Number of hydrogen bonds formed between the $Ti_3C_2T_2$ MXene with different functionalities and Matrimid suggested by molecular simulations.

Sample	Number of H-bonds shorter than 3 Å	Number of H-bonds ranged between 3 and 4 Å
$Ti_3C_2O_2$	3	23
$Ti_3C_2F_2$	4	25
$Ti_3C_2(OH)_2$	4	9
Aminosilane-$Ti_3C_2(OH)_2$	7	20

Table S8. Performance overview of mixed-matrix membranes based on Matrimid 5218 incorporated with various fillers for CO_2/CH_4 separation (pure gases).

Membrane		Operating conditions		Membrane thickness (µm)	Gas separation performance		Ref.
Filler	Optimized filler loading (wt.%)	Pressure (bar)	Temperature (°C)		CO_2 permeability (Barrer)	CO_2/CH_4 ideal selectivity	
Nano-silica	20	2	35	40	9.59	50.74	5
Silica nanoparticles	9	2	25	70–120	23.0	36.5	6
MIL-101	20	10	35	10–15	6.95	56	7
NaY Zeolite	15	2	35	45–55	17.52	43.3	8
SAPO-34	20	10	-	40–70	6.9	67	9
ZIF-11	30	4	35	70	31.36	42.95	10
ZIF-95	30	3	30	25	23.20	58	11
Nonzoned	0.75	1	35	70	11.46	39.52	3
MIL-53	15	3	35	~50	12.43	51.80	12
ZSM-5	10	2.5	35	~40	9.01	30.03	13
MIL-53	30	2	35	50–60	21.6	51.40	14
ZIF-8	30	2	35	50–60	22.10	52.90	14
Cu_3BTC_2	30	2	35	50–60	17.00	42.30	14
MIL-53 (Al)	15	10	35	40–85	6.70	9.40	15
NH_2-MIL-53 (Al)	15	10	35	40–85	9.2	2.10	15
SBMA@CNT	10	2	25	N/A	4.10	97.00	16
Cu-4,40-BPY-HFS	20	2	35	~50	9.88	27.08	17
Cu-BTC	20	2	35	60–100	31.00	36.00	18
POP-2	15	2	35	60–100	17.00	35.20	18
$Ti_3C_2T_x$	2	5	Room temp.	60–70	8.35	33.4	This work
$Ti_3C_2T_x$	5	5	Room temp.	60–70	10.51	50.04	This work
$Ti_3C_2T_x$	8	5	Room temp.	60–70	16.1	42.36	This work
$Ti_3C_2T_x$	10	5	Room temp.	60–70	14.79	36.97	This work
A-$Ti_3C_2T_x$	5	5	Room temp.	60–70	15.29	63.73	This work

Reference

(1) Maleski, K.; Ren, C. E.; Zhao, M.-Q.; Anasori, B.; Gogotsi, Y. Size-Dependent Physical and Electrochemical Properties of Two-Dimensional MXene Flakes. *ACS Appl. Mater. Interfaces.* **2018**, *10* (29), 24491-24498. DOI: 10.1021/acsami.8b04662.
(2) Munir, S.; Rasheed, A.; Rasheed, T.; Ayman, I.; Ajmal, S.; Rehman, A.; Shakir, I.; Agboola, P. O.; Warsi, M. F. Exploring the Influence of Critical Parameters for the Effective Synthesis of High-Quality 2D MXene. *ACS Omega* **2020**, *5* (41), 26845-26854. DOI: 10.1021/acsomega.0c03970.
(3) Li, E.; Chen, Z.; Duan, C.; Yuan, B.; Yan, S.; Luo, X.; Pan, F.; Jiang, Z. Enhanced CO2-capture performance of polyimide-based mixed matrix membranes by incorporating ZnO@MOF nanocomposites. *Separation and Purification Technology* **2022**, *289*, 120714.
(4) Gupta, N. K.; Bae, J.; Kim, S.; Kim, K. S. Fabrication of Zn-MOF/ZnO nanocomposites for room temperature H2S removal: Adsorption, regeneration, and mechanism. *Chemosphere* **2021**, *274*, 129789.
(5) Nezhadmoghadam, E.; Chenar, M. P.; Omidkhah, M.; Nezhadmoghadam, A.; Abedini, R. Aminosilane grafted Matrimid 5218/nano-silica mixed matrix membrane for CO 2/light gases separation. *Korean J. Chem. Eng.* **2018**, *35*, 526-534.
(6) Chen, X. Y.; Razzaz, Z.; Kaliaguine, S.; Rodrigue, D. Mixed matrix membranes based on silica nanoparticles and microcellular polymers for CO2/CH4 separation. *J. Cell. Plast.* **2018**, *54* (2), 309-331.
(7) Naseri, M.; Mousavi, S. F.; Mohammadi, T.; Bakhtiari, O. Synthesis and gas transport performance of MIL-101/Matrimid mixed matrix membranes. *J. Ind. Eng. Chem.* **2015**, *29*, 249-256.
(8) Ebadi Amooghin, A.; Omidkhah, M.; Kargari, A. The effects of aminosilane grafting on NaY zeolite–Matrimid®5218 mixed matrix membranes for CO2/CH4 separation. *Journal of Membrane Science* **2015**, *490*, 364-379.
(9) Peydayesh, M.; Asarehpour, S.; Mohammadi, T.; Bakhtiari, O. Preparation and characterization of SAPO-34 – Matrimid® 5218 mixed matrix membranes for CO2/CH4 separation. *Chem. Eng. Res. Des.* **2013**, *91* (7), 1335-1342.
(10) Yumru, A. B.; Safak Boroglu, M.; Boz, I. ZIF-11/Matrimid® mixed matrix membranes for efficient CO2, CH4, and H2 separations. *Greenhouse Gases: Science and Technology* **2018**, *8* (3), 529-541.
(11) Ilicak, I.; Boroglu, M. S.; Durmus, A.; Boz, I. Influence of ZIF-95 on structure and gas separation properties of polyimide-based mixed matrix membranes. *Journal of Natural Gas Science and Engineering* **2021**, *91*, 103941.
(12) Dorosti, F.; Omidkhah, M.; Abedini, R. Fabrication and characterization of Matrimid/MIL-53 mixed matrix membrane for CO2/CH4 separation. *Chem. Eng. Res. Des.* **2014**, *92* (11), 2439-2448.
(13) Zhang, Y.; Balkus, K. J.; Musselman, I. H.; Ferraris, J. P. Mixed-matrix membranes composed of Matrimid® and mesoporous ZSM-5 nanoparticles. *J. Membr. Sci.* **2008**, *325* (1), 28-39.
(14) Shahid, S.; Nijmeijer, K. Performance and plasticization behavior of polymer–MOF membranes for gas separation at elevated pressures. *J. Membr. Sci.* **2014**, *470*, 166-177.
(15) Chen, X. Y.; Hoang, V.-T.; Rodrigue, D.; Kaliaguine, S. Optimization of continuous phase in amino-functionalized metal–organic framework (MIL-53) based co-polyimide mixed matrix membranes for CO 2/CH 4 separation. *RSC Adv.* **2013**, *3* (46), 24266-24279.

(16) Liu, Y.; Peng, D.; He, G.; Wang, S.; Li, Y.; Wu, H.; Jiang, Z. Enhanced CO2 permeability of membranes by incorporating polyzwitterion@ CNT composite particles into polyimide matrix. *ACS Appl. Mater. Interfaces.* **2014**, *6* (15), 13051-13060.
(17) Zhang, Y.; Musselman, I. H.; Ferraris, J. P.; Balkus Jr, K. J. Gas permeability properties of Matrimid® membranes containing the metal-organic framework Cu–BPY–HFS. *J. Membr. Sci.* **2008**, *313* (1-2), 170-181.
(18) Kanehashi, S.; Chen, G. Q.; Scholes, C. A.; Ozcelik, B.; Hua, C.; Ciddor, L.; Southon, P. D.; D'Alessandro, D. M.; Kentish, S. E. Enhancing gas permeability in mixed matrix membranes through tuning the nanoparticle properties. *J. Membr. Sci.* **2015**, *482*, 49-55.
